# Exercise interventions for self-perceived body image, self-esteem and self-efficacy in women diagnosed with breast cancer: a systematic review with meta-analysis and meta-regressions

**DOI:** 10.1007/s00520-024-08874-9

**Published:** 2024-09-17

**Authors:** María Jesús Casuso-Holgado, Javier Martinez-Calderon, Patricia Martínez-Miranda, María Jesús Muñoz-Fernández, Carlos Bernal-Utrera, Cristina García-Muñoz

**Affiliations:** 1 Departamento de Fisioterapia, Instituto de Biomedicina de Sevilla, IBiS, Universidad de Sevilla, Seville, Spain; 2 CTS 1110: UMSS Research Group, Andalusia, Spain; 3 Department of Physiotherapy, Faculty of Nursing, Physiotherapy and Podiatry, University of Seville, 41009 Seville, Spain; 4 Department of Physiotherapy, University School Francisco Maldonado, Osuna, Spain; 5 Departament of Physiotherapy, University San Isidoro, Seville, Spain; 6 Departamento de Ciencias de La Salud y Biomédicas, Universidad Loyola de Andalucía, Seville, Spain

**Keywords:** Breast neoplasms, Exercise, Body image, Self-efficacy, Self-esteem

## Abstract

**Purpose:**

To synthesise the effectiveness of exercise interventions on self-perceived body image, self-esteem and self-efficacy in women diagnosed with breast cancer who are undergoing or have completed primary adjuvant treatments.

**Methods:**

A systematic review was conducted with meta-analysis and meta-regressions. Five electronic databases were searched from inception to June 2023, and hand searches were performed to explore the reference lists of similar systematic reviews. The established selection criteria were randomised clinical trials that evaluated any type of physical exercise intervention with self-perceived body image, self-esteem and self-efficacy as outcomes. No restrictions were imposed with respect to the control group. Main characteristics were extracted for each study. Meta-analyses, meta-regressions and sensitivity analyses were performed. The certainty of evidence for each outcome was graded using the GRADE approach. The risk of bias was evaluated using the RoB2 Cochrane tool.

**Results:**

Twenty studies, comprising 19 different samples (*n* = 2030), were included. In general, meta-analysis indicated that physical exercise interventions were not superior to controls for improving self-esteem and body image in women diagnosed with breast cancer. However, subgroup meta-analysis showed a significant difference in self-esteem improvement for resistance exercise (SMD = 0.31; 95% CI = 0.07, 0.55; *p* = 0.01; *I*^2^ = 0%) and supervised exercise (SMD = 0.25; 95% CI = 0.08, 0.42; *p* = 0.0004; *I*^2^ = 0%) compared with controls. Self-efficacy results were scarce and controversial. In addition, serious concerns were mainly detected in terms of the risk of bias and indirectness of the evidence, which caused the certainty of evidence to be very low for all outcomes.

**Conclusion:**

Supervised exercise and resistance training appear to be effective exercise modalities for improving self-esteem in women diagnosed with breast cancer. In contrast, exercise interventions are not significantly associated with improvements in body image, while results on self-efficacy are controversial. However, due to the study’s limitations, further research is needed.

**Supplementary Information:**

The online version contains supplementary material available at 10.1007/s00520-024-08874-9.

## Introduction

The latest global cancer statistics declare breast cancer as the second most commonly diagnosed type of cancer worldwide, with 2.3 million new cases detected in 2022 [1]. In addition, breast cancer survival rates have increased in transitioned countries [1], but the physical and psychological morbidity associated with primary cancer therapies remains a public health challenge [2].

Self-perceived body image has been identified in the context of breast cancer as a multidimensional construct referring to the mental image of one’s body together with attitudes towards appearance (e.g. feeling feminine) and sexual functioning [3]. Women with breast cancer often face body changes such as hair loss or weight gain that can have a negative impact on their body image perception [4, 5]. In addition, data from a nationwide survey in the United States revealed that most women feel self-conscious due to scars from their breast cancer surgery and uncomfortable when undressed [6]. All these body changes can also affect women’s self-esteem, which is an individual’s feeling of self-respect and worth [7]. Self-esteem is also known to play a mediating role between body image perception and quality of life in women with breast cancer [8]. Finally, self-efficacy, defined as one’s perceived ability to perform a specific behaviour, is a key component of self-care, as people are only motivated to act if they believe they can influence results [9]. In women with breast cancer, lower levels of perceived self-efficacy to cope with cancer symptoms predict poorer levels of well-being [10, 11].

Over the past decade, the number of scientific publications on exercise in breast cancer has increased exponentially [12], with important findings supporting exercise interventions for the management of cancer-related symptoms [13, 14]. Recent evidence suggests the involvement of endogenous opioids, particularly the mu-opioid system, as a partial mediator of the effects of regular exercise on mood elevation [15], although evidence-based studies in women with breast cancer are needed [16]. Preliminary evidence appears to support the benefit of exercise interventions on body image [16, 17], while the effect of exercise on self-esteem remains unclear [16]. Increases in physical activity lead to increases in self-efficacy, which has a positive impact on self-esteem [18], but the effect of exercise on general self-efficacy (referred to daily living tasks) or specific self-efficacy behaviours (e.g. exercise self-efficacy, symptom management self-efficacy) has not been previously synthesised in this population. Thus, this systematic review with meta-analysis aimed to synthetise the effectiveness of exercise interventions on self-perceived body image, self-esteem and self-efficacy in women diagnosed with breast cancer who are undergoing or have completed primary adjuvant treatments.

## Methods

This systematic review was registered in PROSPERO (CRD42023393852) and has been conducted following the 2020 Preferred Reporting Items for Systematic Reviews and Meta-Analyses (PRISMA) [19] statement and the PRISMA checklist for abstracts [20].

### Data sources and search strategy

One reviewer (JM-C) searched in PubMed, Embase, PsycINFO, CINHAL and SPORTDiscus databases from their inception to June 2023. Among others, Medical Subjects Heading (MeSH) terms such as “exercise”, “training”, “breast”, “cancer” and “body-image” were used, adapting the search strategy to the different databases’ requirements. The type of document and the language of the publication were used as search filters. The full search strategy for each database is reported in Table S1 (Supplementary Table S1).

Another reviewer (MJM-F) developed a manual search by checking the reference lists of similar systematic reviews.

### Eligibility criteria

The review question was defined using the PICOS framework [21], (P; population; I, intervention; C, comparison; O, outcomes; S, study design) as follows: *Can exercise practice influence self-perceived body image, self-esteem and self-efficacy in women diagnosed with breast cancer?*

The inclusion criteria were:

- (P): Women over 18 years old with breast cancer diagnosis (stages I–IV). They can be undergoing primary adjuvant treatments (e.g. chemotherapy or radiation therapy) or have finalised them (survivorship phase) [22]. As it is common in breast cancer research, women with hormonal therapy were included as they are not considered to be “under primary treatment” [23].
- (I): Any type of physical exercise as defined by the World Health Organization (WHO): *A subcategory of physical activity that is planned, structured, repetitive and purposeful in the sense that the improvement or maintenance of one or more components of physical fitness is the objective *[24]*.* For inclusion, a clear exercise intervention prescription based on duration, frequency, and/or intensity had to be set.
- (C): No restrictions were imposed regarding the control group.
- (O): Self-perceived body image, self-esteem, self-efficacy; only quantitative measures using validated scales or questionnaires were included.
- (S): Randomised controlled trials (RCTs) written in English or Spanish. Pilot or feasibility RCTs were also included.

The exclusion criteria were studies involving subjects affected by other types of cancer without subdivision of results, prehabilitation exercises and multimodal interventions that combine exercise programmes with other non-exercise interventions. RCTs based on general exercise recommendations were also excluded.

### Study selection

Duplicates were removed using the Mendeley desktop citation management software v1.19.8 and manually checked by one reviewer (MJM-F). This reviewer also screened titles and abstracts of all records according to the abovementioned PICOS question. The full texts were evaluated when abstracts seemed eligible or when abstracts were unavailable. In case of doubt, a second reviewer (MJC-H) was consulted to determine if a specific study was or was not included, applying the eligibility criteria. This step was necessary for 17 studies (Supplementary Table S2).

### Data synthesis

Data were extracted by two reviewers (PM-M and CB-U) and revised by a third reviewer (MJC-H). The following data were extracted for each study, when possible: first author, year of publication, country, risk of bias assessment, population’s details such as sample size by groups and age, intervention details (exercise modality, the number and duration of sessions), control group details, outcomes (instruments and points of assessment), and main findings. This information was synthesised and displayed in a table of characteristics.

Afterward, quantitative data was extracted by the same three reviewers in a Microsoft Excel spreadsheet (v. 2007). Subsequently, two reviewers (CG-M and MJC-H) used the R studio software (v. 4.1.1) with the packages of meta (v.5.1–1) [25], metafor (v.3.0–2) [26] and dmetar (v.0.0.9000) [27] to conduct each meta-analysis. Meta-analyses were carried out according to the outcome of interest and the assessment point (immediately after intervention). Two corresponding authors were contacted several times for data requirements, but no answer was provided [28, 29]; four RCTs did not report sufficiently homogenous data to be synthesised [23, 30–32], and another study did not report the corresponding author’s contact [33]. Moreover, two studies shared the same sample, and they were grouped [34, 35]. As a result, a total of 12 RCTs were included in our meta-analysis.

A random-effect model was used assuming the presence of heterogeneity among the RCTs. Data were pooled with an inverse variance weighting method, and standard mean differences (SMDs) were estimated using the Hedge’s g method. The sizes of the Hedge’s *g* effect can be classified into small effect (*g* = 0.2), medium effect (*g* = 0.5) or large effect (*g* = 0.8). Heterogeneity among clinical trials was assessed using *I*^2^ statistics (notable heterogeneity when *I*^2^ > 50%). Forest plots were designed to report the results of each meta-analysis. Sensitivity analyses were developed to detect outliers or influential cases by an exploratory analysis of the data (doi plot, leave-one-out methods and baujat plot). If one study was detected as an outlier or influential case, it was removed from the meta-analysis. The prediction interval was added to the forest plots when the meta-analysis included at least three trials and accounts for the heterogeneity between the trials to assess the probability if true treatment effects can be expected in future settings [36]. Subgroup analyses were performed to explore possible sources of heterogeneity related to exercise programmes characteristics: type of exercise (endurance, resistance, multimodal or mind–body exercises), group versus individual training, exercise supervision and risk of bias. We considered Pilates as a resistance exercise modality [37, 38].

Meta-regressions were performed to explore if pooled effect size was influenced by the following predictors at both the person level and the study level [39]: age, sample size and exercise prescription parameters (minutes/session, number of sessions, weeks of intervention). Covariates were evaluated if they were reported at least in three studies and were not iterative.

In addition, publication bias and the possible presence of small-study effects were tested using a funnel plot and Egger’s test [40]. The latter is conducted when at least there are three studies and confirms publication bias when *p* < 0.05.

### Risk of *bias* assessment

The risk of bias was independently evaluated by two reviewers (CB-U and MJM-F) using the Cochrane Risk of Bias Tool for Randomised Trials (RoB-2) [41]. It was not necessary to consult a third reviewer (MJC-H) as consensus was reached in all cases. The RoB-2 tool is based on five different domains: randomisation process; deviations from intended interventions; missing outcome data, measurement tools; and selection of reported findings. Within each domain, several “signaling questions” need to be answered to elicit relevant information. The overall risk of bias can be judged as “high” or “low” or may indicate “some concerns”. Before pooling the results of the independent assessments, the percentage of agreement between CB-U and MJM-F was calculated considering the number of items rated with the same score after counting all items.

### The certainty of evidence

The certainty of evidence for each outcome was graded using the Grading of Recommendations, Assessment, Development and Evaluation (GRADE) system [42]. The evidence of RCTs in GRADE begins as high evidence and can be downgraded one or two levels, depending on the presence of serious (− 1 level) or very serious (− 2 levels) concerns in terms of risk of bias, inconsistencies of the findings, indirectness of the evidence, imprecision of the results and publication bias. Two independent reviewers (CG-M and MJC-H) rated the overall evidence as follows: high, it is very likely that the true effect is like the estimated effect; moderate, the true effect is probably close to the estimated effect; low, denoting that the true effect may be considerably different from the estimated effect; and very low, when any estimate of effect is very uncertain.

### Reported description of the interventions

The replicability of the interventions was checked by one reviewer (PM-M) using the Template for Intervention Description and Replication (TIDieR) checklist, which is based on 12 items to assess if the interventions were reported in sufficient detail to be replicated (e.g. what materials and procedures, who provided the intervention, where, when and how much) [43].

### Protocol deviation

Some deviations from the review protocol arose. The search strategy was updated until June 2023 instead of February 2023. In addition, it was not possible to conduct a meta-analysis for self-efficacy due to the heterogeneity in the conceptualization of this outcome.

## Results

### Study selection

**Fig. 1:**
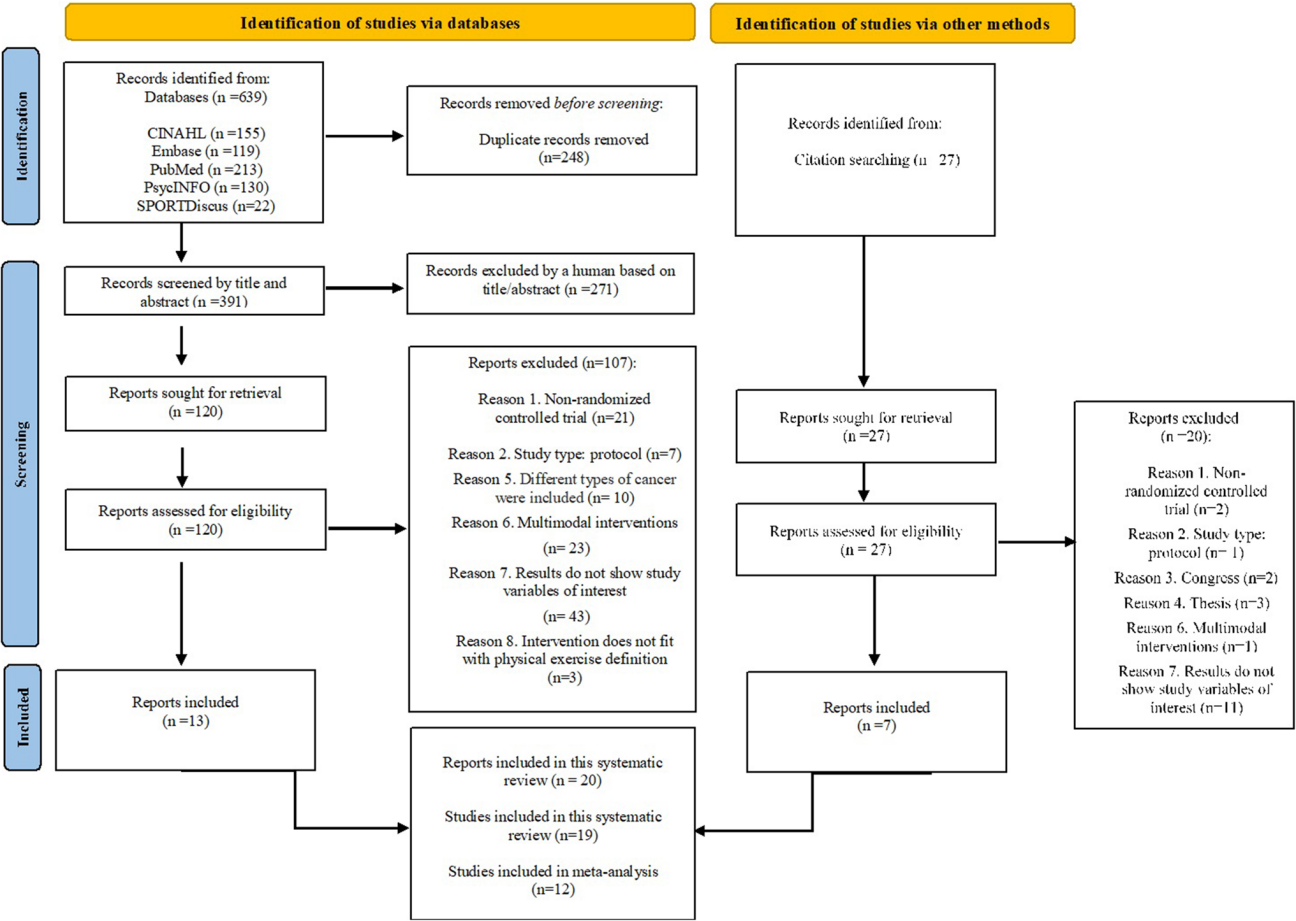
PRISMA flow diagram

A total of 639 records from databases and 27 from manual searching were identified of which 146 were full-text retrieved. Finally, 20 records comprising 19 RCTs were included (Fig. 1). Supplementary Table S2 contains a full list of the records excluded in the last step (*n* = 127) and the reasons.

### General study description

A total of 2030 participants were included in this systematic review, and 1824 of them were included in our meta-analysis. Most of the RCTs included participants in the survivorship phase, with only five of them investigating the effects of exercise interventions in self-perceived body image [44, 45], self-esteem [29, 34, 35] and exercise self-efficacy [32] during primary adjuvant treatments. Seven of the 19 RCTs focused on endurance training [30, 31, 33, 46–48, 54], three on muscle resistance [45, 49, 50], four combined both resistance and endurance exercises [29, 51, 52] and three studied different types of mind–body exercises [23, 28, 32]. Moreover, two studies included resistance, endurance and multimodal exercises in several groups [34, 35, 53]. Most of the exercise interventions were supervised; the intervention period ranged from 4 to 53 weeks, with a minimum of 4 weeks and 12 sessions. In most RCTs, exercise training was compared with no intervention [23, 29–31, 33–35, 46–48, 51, 52, 54], but also with flexibility or muscle relaxation exercises [45, 53], sham flexibility [44] and educational/counselling interventions [28, 49, 50] or with general recommendations to exercise [32].

Self-perceived body image was measured heterogeneously across studies using a total of five different body image instruments: The European Organization for Research and Treatment of Cancer Breast Cancer-Specific Quality of Life Questionnaire (EORTC-BRE23) body image subscale [44, 45, 48, 51] which includes items on body attractiveness, body acceptance and femininity; the Body Image After Breast Cancer Questionnaire (BIBCQ) body stigma subscale [50], which assesses impairment of femininity and attractiveness; the Body Image Questionnaire (BIQ) individual subscale [47], assessing body satisfaction; the Physical Self-perception Profile (PSPP) attractive body domain [53], which focuses on self-perceptions of body attractiveness; and the Body Image and Relationships Scale (BIRS) appearance and sexuality subscale [52], which assess both self-perceptions of appearance and sexual functioning. In contrast, self-esteem was measured uniformly using the Rosenberg Self-Esteem Scale [28, 29, 33–35, 46, 49, 50, 53, 54], except in one study [30]. Finally, self-efficacy was assessed using the German Self-efficacy Questionnaire [23], the Self-Efficacy and Physical Activity Scale [31] and the Self-efficacy Questionnaire [32]. A detailed description of the studies is reported in Supplementary Table S3.

### Risk of *bias* assessment

**Fig. 2:**
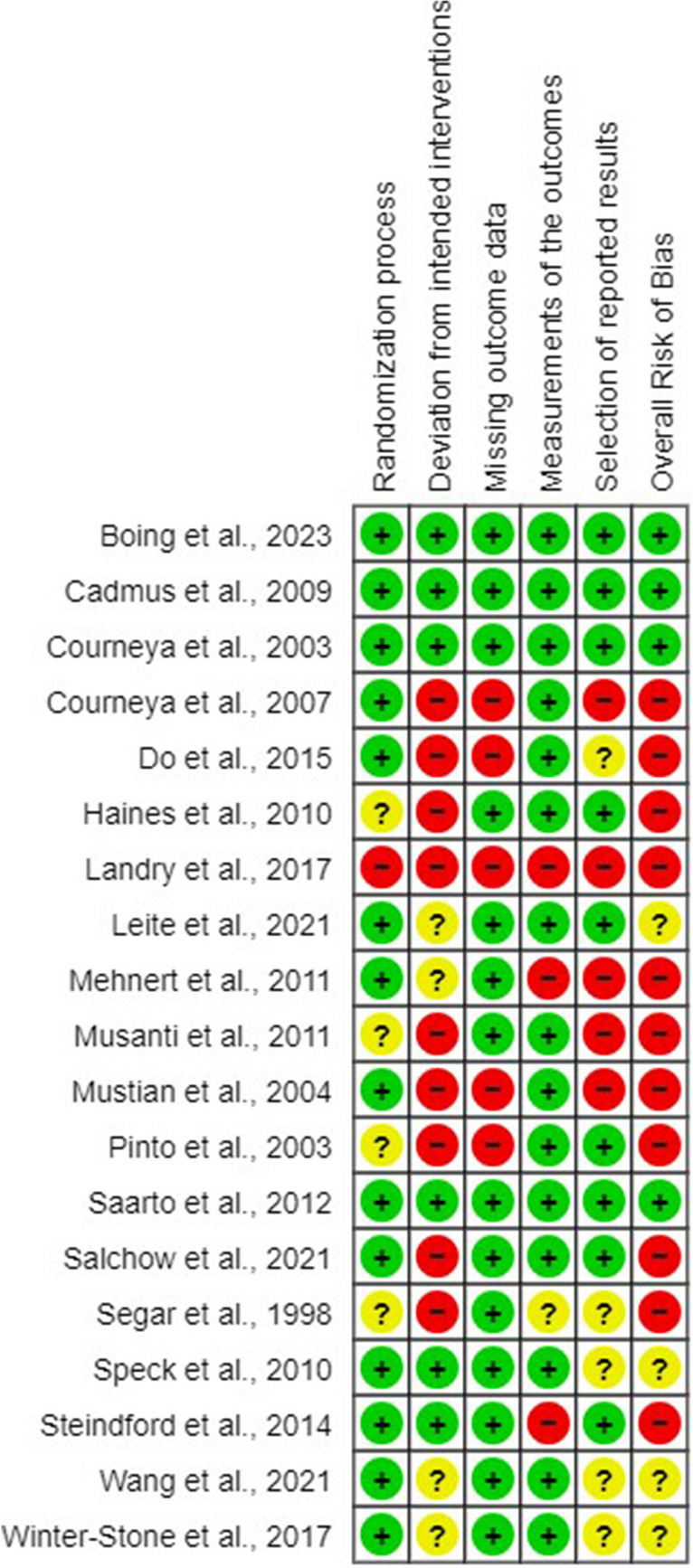
RoB graph

Eleven RCTs were judged to have an overall high risk of bias [23, 28–30, 33–35, 44, 45, 47, 51, 53]. Bias due to deviations from intended interventions and from the selection of the reported results were the most frequently observed (Fig. 2). Inter-rater reliability was 82%.

### Replication of the interventions

In general, most of the RCTs described in detail the interventions, particularly item 4, “What: procedure” (89.5%); item 6, “How” (94.7%); and item 8, “When and How much” (89.5%). On the contrary, item 10 “Modifications” was scarcely reported (10.5%) (Supplementary Table S4).

### The certainty of evidence (GRADE)

Serious concerns were mainly detected in terms of the risk of bias and indirectness of the evidence, which caused the certainty of evidence to be judged as very low for all outcomes with an inter-rater reliability of 86% (Supplementary Table S5).

### Synthesis of the evidence: exercise interventions on body image (GRADE: very low evidence)

**Fig. 3:**
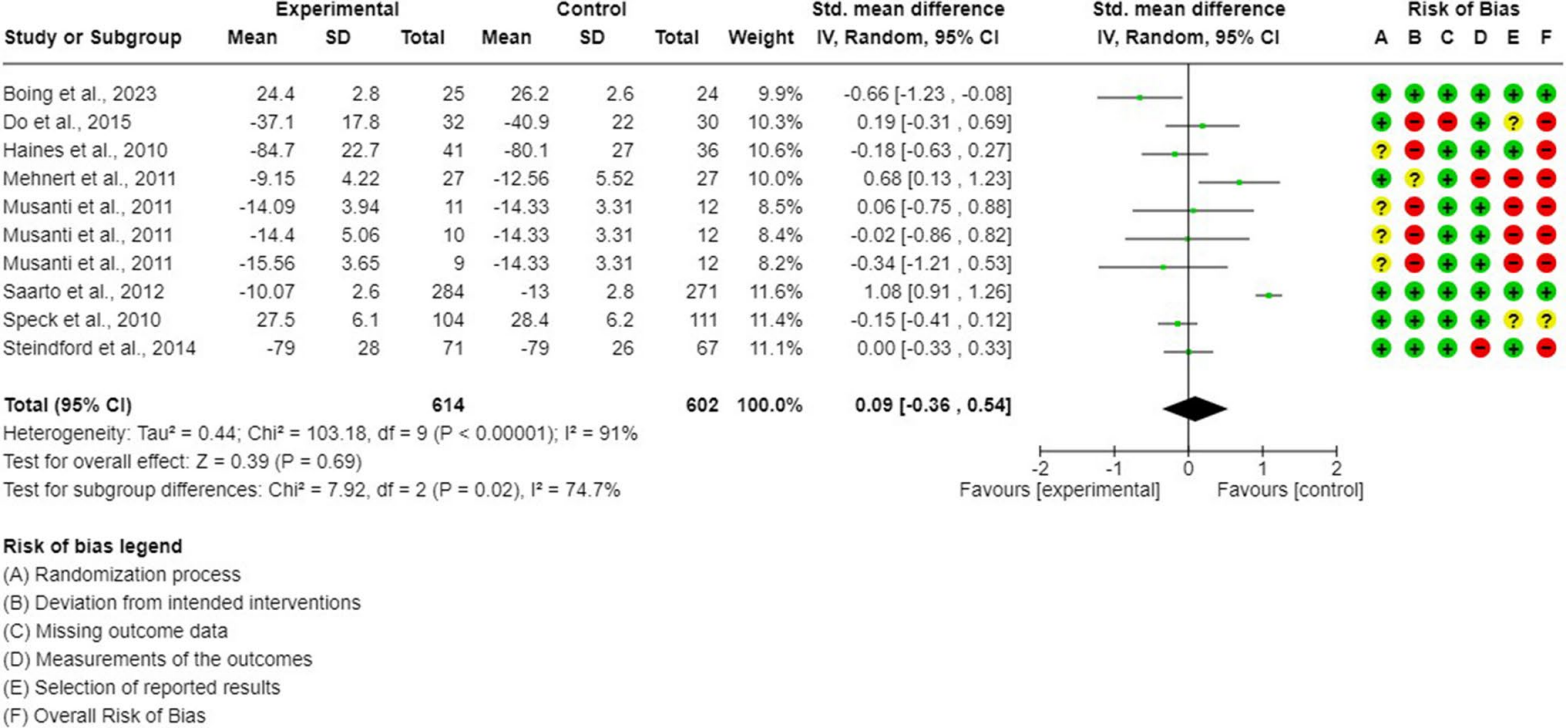
Body image meta-analysis

**Fig. 4:**
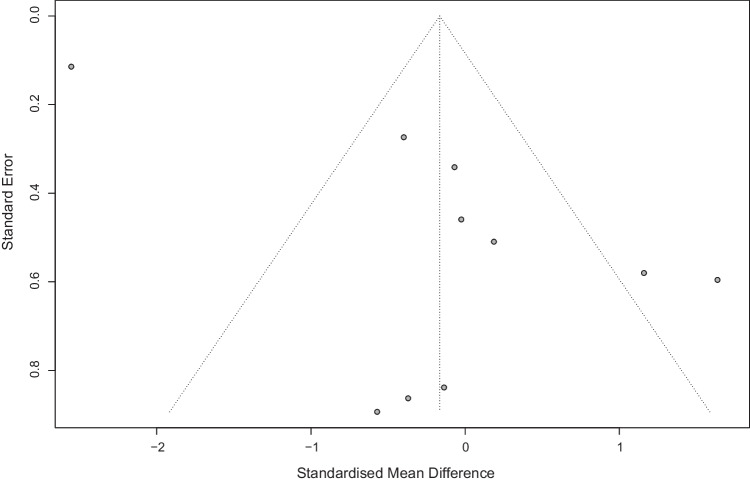
Funnel plot. Body image

Eight RCTs (10 arms) were included in a meta-analysis of self-perceived body image [44, 45, 47, 48, 50–53]. In general, no differences were shown between exercise-based interventions and control interventions (SMD = 0.09; 95% CI =  − 0.36, 0.54; *p* = 0.69; *I*^2^ = 91%) (Fig. 3). The sensitivity analysis showed how heterogeneity decreased (*I*^2^ = 40%) after excluding one outlier [48], but exercise interventions remained not superior to controls (SMD =  − 0.04; 95% CI =  − 0.26, 0.18; *p* = 0.72) (Supplementary Figure S6). Publication bias was detected (Egger’s test *p* = 0.02; Fig. 4).

### Subgroup *meta*-analysis of body image

Subgroup meta-analysis revealed the existence of subgroup differences for exercise type (Supplementary File 1, Table S7). When data from different modalities of the exercise was pooled separately, it was shown that endurance interventions were significantly inferior to controls for self-perceived body image improvement (SMD = 0.70; 95% CI = 0.14, 1.27; *p* = 0.01; *I*^2^ = 74%), while no differences were observed between resistance or multimodal training and controls (Supplementary Figure S8). No other subgroup differences were observed based on exercise intervention characteristics (supervision, group) or the risk of bias in the included studies (Supplementary Figures S9–S11).

### *Meta*-regression analysis of body image

Based on meta-regression analysis (Supplementary Table S12), the overall effect size for self-perceived body image was significantly influenced by sample size (*β* =  − 0.01; *p* < 0.001), number of experimental sessions (*β* =  − 0.02; *p* = 0.01), and weeks of intervention (*β* =  − 0.04; *p* = 0.04) (Supplementary Figures S13–S15).

### Synthesis of the evidence: exercise interventions on self-esteem (GRADE: very low evidence)

**Fig. 5:**
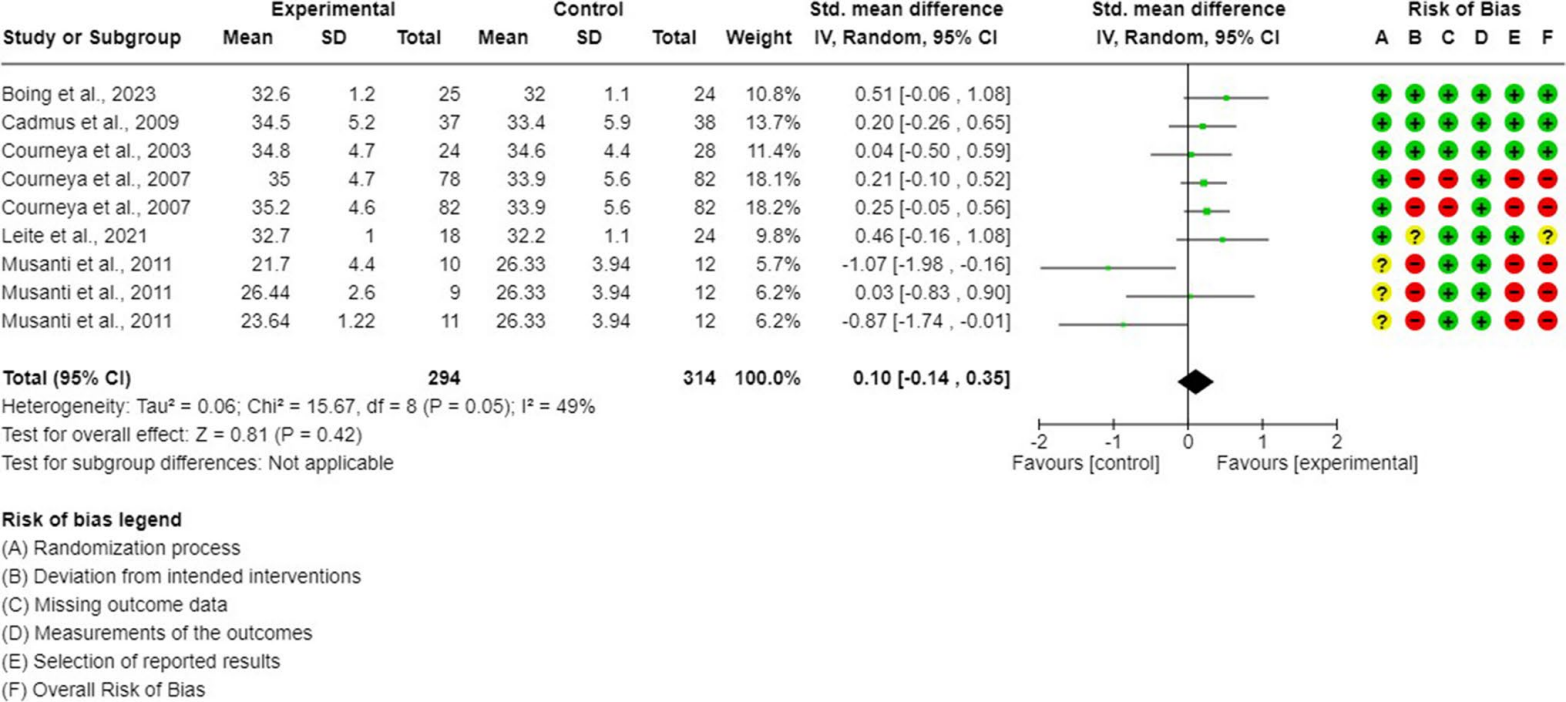
Self-esteem meta-analysis

**Fig. 6:**
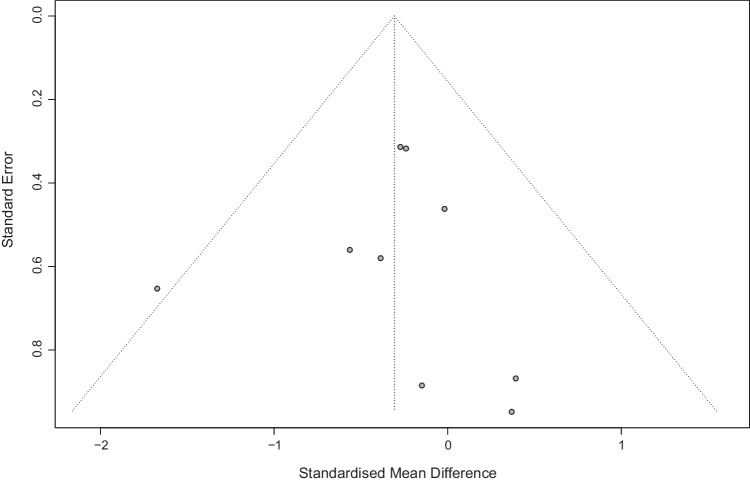
Funnel plot. Self-esteem

Data from six RCTs (9 arms) was pooled in a meta-analysis of self-esteem [34, 35, 46, 49, 50, 53, 54]. In general, no differences were shown between exercise-based interventions and control interventions (SMD = 0.10; 95% CI =  − 0.14, 0.35; *p* = 0.42; *I*^2^ = 49%) (Fig. 5). The sensitivity analysis showed how heterogeneity decreased (*I*^2^ = 0%) after excluding endurance and multimodal interventions from one outlier [53], and exercise interventions were significantly superior to control interventions in improving self-esteem (SMD = 0.24; 95% CI = 0.08, 0.41; *p* = 0.004) (Supplementary Figure S16). Publications bias was not detected (Egger’s test *p* = 0.97; Fig. 6).

### Subgroup meta-analysis of self-esteem

Subgroup meta-analysis showed the existence of subgroup differences for exercise type and exercise supervision (Supplementary File 1, Table S17). Resistance interventions were significantly superior to control conditions for self-esteem improvement (SMD = 0.31; 95% CI = 0.07, 0.55; *p* = 0.01; *I*^2^ = 0%), while no differences were observed between endurance or multimodal training and controls (Supplementary Figure S18).

Moreover, supervised exercise programmes were also superior to controls (SMD = 0.25; 95% CI = 0.08, 0.42; *p* = 0.0004; *I*^2^ = 0%), but no differences were observed between unsupervised exercise interventions and controls. No other subgroup differences were observed (Supplementary Figures S19–S21).

### Meta-regression analysis of self-esteem

The overall effect size for self-esteem seems to be not significantly influenced by any of the outcomes included in our meta-regression (Supplementary Table S22).

### Exercise interventions on self-efficacy

Self-efficacy was investigated in three RCTs [23, 31, 32]. However, while Salchow et al. [23] investigated the effect of Kyusho Jitsu on general self-efficacy, Wang et al. [31] and Winter-Stone et al. [32] investigated the effect of endurance exercise and yoga on exercise self-efficacy, respectively. This lack of homogeneity in the conceptual outcome assessed and the exercise type applied prevented us from pooling these data in the same meta-analysis. Concerning the observed results, only Wang et al. [31] reported significant differences in favour of the experimental group (*p* = 0.047).

## Discussion

This systematic review with meta-analysis aimed to summarise the effect of exercise interventions on self-perceived body image, self-esteem and self-efficacy in women diagnosed with breast cancer. In general, our pooled results suggest that exercise interventions are not superior to controls for improving body image but seem to be more effective than control conditions for improving self-esteem, particularly when supervised and resistance programmes are applied. Group exercise interventions did not show advantages over individual training, which is consistent with findings on quality of life in this population [55].

Previous research has found a significant association between exercise interventions and body image improvement [16], which is not supported by our results. We hypothesise that differences in intervention characteristics may explain our results, as meta-regression analysis showed that the pooled effect size was influenced by some exercise prescription parameters (i.e. number of sessions and weeks of intervention). In addition, body image in women diagnosed with breast cancer is considered to be a complex issue involving the affective (e.g. feeling attractive), behavioural (e.g. avoiding certain clothes) and cognitive domains (e.g. accepting body changes) [56–58]. We hypothesise that combining exercise with other interventions (e.g. cognitive behavioural therapy) may be the way forward in future research to better manage body image concerns in women with breast cancer.

Subgroup meta-analyses suggest that resistance exercise and supervised modalities are effective interventions for promoting self-esteem in women diagnosed with breast cancer. Most of the preliminary research on this topic has not examined the effect of different exercise modalities on self-esteem separately, with controversial results [16, 59, 60]. Furthermore, in contrast to our results, Han et al. [61] concluded that resistance exercise was not associated with significant benefits on self-esteem in women with breast cancer, although the number of studies included in their analysis was small. The mechanisms by which resistance training might improve self-esteem have been investigated in healthy populations and remain unclear. Cognitive changes have been suggested as a possible explanation for this relationship [62], but studies in women with breast cancer are needed. In addition, we hypothesise that tailored exercise programmes delivered face-to-face by a health professional may be an effective strategy for improving self-efficacy, which will have a mediating role on self-esteem and explain why supervised exercise modalities are effective in improving self-esteem [63]. However, the association between exercise interventions and self-efficacy in women diagnosed with breast cancer has been poorly investigated [23, 31, 32] and this is the first systematic review to synthesise this relationship, with controversial findings observed.

Self-efficacy is an important construct in human behaviour theories because of its high predictive value [9]. It is the most frequently identified psychosocial determinant of physical activity behaviour which is known to play a role in the maintenance of health behaviours over time [63]. A panel model demonstrated that women with breast cancer with higher levels of physical activity perceived themselves as more self-efficacious, which had a mediating effect on physical self-worth and global self-esteem [18]. Therefore, it would be important for future research to clarify which exercise characteristics could help to promote self-efficacy, which is expected to have a positive impact on self-esteem and well-being [10, 11, 62].

### Limitations

First, although we only included interventions based on a clear exercise prescription, heterogeneity between trials may have influenced our results. Secondly, methodological flaws were found in most of the trials analysed, which force us to interpret our results with caution. Third, the scarce number of studies investigating the effects of exercise training on self-efficacy beliefs prevented us from pooling data for this outcome; for the same reason, the small number of trials that included women during primary adjuvant treatments or mind–body exercise modalities did not allow us to perform these subgroup analyses, so no conclusions can be drawn. Finally, our results may have been influenced by the different rates of adherence to exercise prescription that were observed between trials.

### Clinical implications

The main clinical implication of this systematic review is that women diagnosed with breast cancer may benefit from resistance exercise and supervised training to improve their self-esteem. Experts in oncology exercise could recommend tailored supervised exercise programmes and resistance training (including Pilates) as they appear to be safe and effective for this purpose. At the moment, our findings do not allow us to recommend any exercise modality over other interventions to improve body image in this population. However, we need to be cautious about interpreting these results, as the overall certainty of evidence was very low.

### Future agenda

Future research could focus on clarifying which parameters of exercise prescription might influence body image in women with breast cancer, when used alone or in combination with other therapies. It would be also of interest to clarify the relationship between exercise practice, self-efficacy and self-esteem in this population. In addition, qualitative research could help clinicians guide patients to an exercise programme that best suits their needs and preferences, an important aspect of improving exercise adherence [64]. Overall, more high-quality studies are needed and the quality of certainty of evidence needs to be improved.

## Conclusion

Based on the available evidence, exercise interventions do not appear to be significantly associated with improvements in body image. In contrast, supervised exercise and resistance training appear to be superior to controls for improving self-esteem in women diagnosed with breast cancer. Finally, self-efficacy has only been tentatively studied, with controversial results reported, so it is not possible to draw any conclusion.

## Supplementary Information

Below is the link to the electronic supplementary material.

Supplementary file1 (DOCX 2211 KB)

## Data Availability

No datasets were generated or analysed during the current study.
