# Supplementary material for: Exercise interventions for self-perceived body image, self-esteem and self-efficacy in women diagnosed with breast cancer: a systematic review with meta-analysis and meta-regressions
Source: Support Care Cancer. 2024 Sep 17;32(10):665. doi: 10.1007/s00520-024-08874-9 (PMC11413083; doi:10.1007/s00520-024-08874-9)
Supplement: Supplementary file 1 — Supplementary file1 (DOCX 2211 KB) [file 520_2024_8874_MOESM1_ESM.docx]

**SUPPLEMENTARY FILE.** Exercise interventions for self-perceived body image, self-esteem and self-efficacy in women diagnosed with breast cancer: A systematic review wit meta-analysis.

**Table S1.** Search Strategies**.**

| **CINAHL**  AB (exercise OR physical-activity OR training OR yoga OR yogic OR pranayama OR tai-chi OR taichi OR qigong OR qi-gong OR Tai-Ji OR Tai OR T'ai OR kung OR chung OR Baduanjin OR Wuqinxi OR Liuzijue OR Yijinjing OR mind-body OR mindful OR qi-training OR gong OR taijiquan) AND TX (body-image OR self-image OR self-esteem OR self-efficacy) AND AB (breast) AND (MH "Neoplasms+" OR AB (oncology OR cancer OR palliative OR metasta*)) AND AB (random*)  Search modes - Boolean/Phrase.  Search filter: source: academic publications.  Search filter: language: English or Spanish.  **(studies retrieved = 155)** |
| --- |
| **Embase**  (exercise:ti,ab OR 'physical activity':ti,ab OR training:ti,ab OR yoga:ti,ab OR yogic:ti,ab OR pranayama:ti,ab OR 'tai chi':ti,ab OR taichi:ti,ab OR qigong:ti,ab OR 'qi gong':ti,ab OR 'tai ji':ti,ab OR tai:ti,ab OR t`ai:ti,ab OR kung:ti,ab OR chung:ti,ab OR baduanjin:ti,ab OR wuqinxi:ti,ab OR liuzijue:ti,ab OR yijinjing:ti,ab OR 'mind body':ti,ab OR mindful:ti,ab OR 'qi training':ti,ab OR gong:ti,ab OR taijiquan:ti,ab) AND (body-image OR self-image OR self-esteem OR self-efficacy) AND (breast:ti,ab) AND ('neoplasm'/exp OR 'oncology'/exp OR 'metastasis'/exp OR 'cancer'/exp OR palliative:ab,ti OR metasta*:ab,ti) AND (random*:ti,ab)  Search filter: publication type: review OR article OR article in press.  Search filter: language: English OR Spanish.  **(studies retrieved = 119)** |
| **PsycINFO**  abstract((exercise OR physical activity OR training OR yoga OR yogic OR pranayama OR tai-chi OR taichi OR qigong OR qi-gong OR Tai-Ji OR Tai OR T'ai OR kung OR chung OR Baduanjin OR Wuqinxi OR Liuzijue OR Yijinjing OR mind-body OR mindful OR qi-training OR gong OR taijiquan) ) AND (body-image OR self-image OR self-esteem OR self-efficacy) AND (MAINSUBJECT.EXACT.EXPLODE "Neoplasms" OR abstract(cancer OR oncology OR palliative OR metasta*)) AND abstract(random*)  Search filter: type of document: Scientific journals.  Search filter: language: English or Spanish.  **(studies retrieved = 130)** |
| **PubMed**  (exercise [tiab] OR physical-activity [tiab] OR training [tiab] OR yoga [tiab] OR yogic [tiab] OR pranayama [tiab] OR tai-chi [tiab] OR taichi [tiab] OR qigong [tiab] OR qi-gong [tiab] OR Tai-Ji [tiab] OR Tai [tiab] OR T'ai [tiab] OR kung [tiab] OR chung [tiab] OR Baduanjin [tiab] OR Wuqinxi [tiab] OR Liuzijue [tiab] OR Yijinjing [tiab] OR mind-body [tiab] OR mindful [tiab] OR qi-training [tiab] OR gong [tiab] OR taijiquan [tiab]) AND (body-image [all] OR self-image [all] OR self-esteem [all] OR self-efficacy [all]) AND (breast [tiab]) AND (neoplasms [mh] OR cancer [tiab] OR oncology [tiab] OR palliative [tiab] OR metasta* [tiab]) AND (random* [tiab])  Search filter: language: English OR Spanish.  **(studies retrieved = 213)** |
| **SPORTDiscus**  AB (exercise OR physical-activity OR training OR yoga OR yogic OR pranayama OR tai-chi OR taichi OR qigong OR qi-gong OR Tai-Ji OR Tai OR T'ai OR kung OR chung OR Baduanjin OR Wuqinxi OR Liuzijue OR Yijinjing OR mind-body OR mindful OR qi-training OR gong OR taijiquan) AND TX (body-image OR self-image OR self-esteem OR self-efficacy) AND AB (breast) AND (MH "Tumors+" OR AB (oncology OR cancer OR palliative OR metasta*)) AND AB (random*)  Search modes - Boolean/Phrase.  **(studies retrieved = 22)** |

**Table S2.** List of excluded records with reasons.

| **Reason 1. Non-randomized controlled trial** | |
| --- | --- |
|  | A E. Speed-Andrews, C. Stevinson, L.J. Belanger, J.J. Mirus, K.S. Courneya. Pilot Evaluation of an Iyengar Yoga Program for Breast Cancer Survivors. Cancer Nursing, Vol. 33, No. 5, 2010. DOI: 10.1097/NCC.0b013e3181cfb55a. PMID: **20467310.** |
|  | M. Berretta, B.A. Facchini, D. Garozzo, V. Necci, R. Taibi, C. Torrisi, G. Ficarra, A. Bitto. Adapted physical activity for breast cancer patients: shared considerations with two Olympic and world Italian sports champions. European Review for Medical and Pharmacological Sciences. 2022; 26 (15): 5393-5398. doi:10.26355/eurrev_202208_29406. PMID: **35993633.** |
|  | Stephanie L. Fowler, William M. P. Klein, Linda Ball, Jaclyn McGuire, Graham A. Colditz, Erika A. Waters. Using an Internet-Based Breast Cancer Risk Assessment Tool to Improve Social-Cognitive Precursors of Physical Activity. Med Decis Making. 2017 Aug;37(6):657-669. doi: 10.1177/0272989X17699835. PMID: **28363033.** PMCID: PMC5498243 |
|  | Anne Marie Lunde Husebø, Bjørg Karlsen, Helen Allan, Jon Arne Søreide and Edvin Bru. Factors perceived to influence exercise adherence in women with breast cancer participating in an exercise programme during adjuvant chemotherapy: a focus group study. J Clin Nurs. 2015 Feb;24(3-4):500-10. doi: 10.1111/jocn.12633. PMID: **24890796.** |
|  | Karen K. Swenson, Mary Jo Nissen, Susan J. Henly. Physical Activity in Women Receiving Chemotherapy for Breast Cancer: Adherence to a Walking Intervention. Oncol Nurs Forum. 2010 May;37(3):321-30. doi: 10.1188/10.ONF.321-330. PMID: **2043921.** |
|  | Minna-Liisa Luoma, Liisa Hakamies-Blomqvist, Carl Blomqvist, Riku Nikander, Mila Gustavsson-Lilius, Tiina Saarto. Experiences of Breast Cancer Survivors Participating in a Tailored Exercise Intervention −A Qualitative Study. Anticancer Res. 2014 Mar;34(3):1193-9. PMID: **24596359.** |
|  | Carol D. Ott, Ada M. Lindsey, Nancy L. Waltman, Gloria J. Gross, Janice J. Twiss, Kris Berg, Patricia L. Brisco, Sharon Henricksen. Facilitative Strategies, Psychological Factors, and Strength/Weight Training Behaviors in Breast Cancer Survivors Who Are at Risk for Osteoporosis. Orthop Nurs. 2004 Jan-Feb;23(1):45-52. doi: 10.1097/00006416-200401000-00013. PMID: **14999952.** |
|  | Siobhan M. Phillips, Gillian R. Lloyd, Elizabeth A. Awick, Edward McAuley. Relationship between self-reported and objectively measured physical activity and subjective memory impairment in breast cancer survivors: role of self-efficacy, fatigue and distress. Psychooncology. 2017 Sep;26(9):1390-1399. doi: 10.1002/pon.4156. PMID: **27388973.** PMCID: PMC5507740. |
|  | Siobhan M. Phillips, Edward McAuley. Social cognitive influences on physical activity participation in long-term breast cancer survivors. Psychooncology. 2013 Apr;22(4):783-91. doi: 10.1002/pon.3074. Epub 2012 Mar 26. PMID: **22451113.** PMCID: PMC4097864. |
|  | Siobhan M. Phillips, Edward McAuley. Physical Activity and Fatigue in Breast Cancer Survivors: A Panel Model Examining the Role of Self-efficacy and Depression. Cancer Epidemiol Biomarkers Prev. 2013 May;22(5):773-81. doi: 10.1158/1055-9965.EPI-12-0983. PMID: **23456557.** PMCID: PMC3650084. |
|  | David S. Shannahoff-Khalsa. Kundalini Yoga Meditation Techniques for Psycho-oncology and as Potential Therapies for Cancer. Integr Cancer Ther. 2005 Mar;4(1):87-100. doi: 10.1177/1534735404273841. PMID: **15695478.** |
|  | Daniela L. Stan, Sarah M. Rausch, Kathleen Sundt, Andrea L. Cheville, James W. Youdas, David A. Krause, Judy C. Boughey, Molly F. Walsh, Stephen S. Cha, MS, Sandhya Pruthi. Pilates for Breast Cancer Survivors: Impact on Physical Parameters and Quality of Life After Mastectomy. Clin J Oncol Nurs. 2012 Apr;16(2):131-41. doi: 10.1188/12.CJON.131-141. PMID: **22459522.** |
|  | Nadine Ungar, Fiona S Rupprecht, Karen Steindorf, Joachim Wiskemann, Monika Sieverding. Worse or even better than expected? Outcome expectancies and behavioral experiences in the context of physical activity among cancer patients. J Health Psychol. 2021 Apr;26(5):659-671. doi: 10.1177/1359105319832345. PMID: **30854903.** |
|  | [Lisa Cadmus-Bertram](https://pubmed.ncbi.nlm.nih.gov/?size=100&term=Cadmus-Bertram+L&cauthor_id=23663078), [Alyson J Littman](https://pubmed.ncbi.nlm.nih.gov/?size=100&term=Littman+AJ&cauthor_id=23663078), [Cornelia M Ulrich](https://pubmed.ncbi.nlm.nih.gov/?size=100&term=Ulrich+CM&cauthor_id=23663078), [Rachael Stovall](https://pubmed.ncbi.nlm.nih.gov/?size=100&term=Stovall+R&cauthor_id=23663078), [Rachel M Ceballos](https://pubmed.ncbi.nlm.nih.gov/?size=100&term=Ceballos+RM&cauthor_id=23663078), [Bonnie A McGregor](https://pubmed.ncbi.nlm.nih.gov/?size=100&term=McGregor+BA&cauthor_id=23663078), [Ching-Yun Wang](https://pubmed.ncbi.nlm.nih.gov/?size=100&term=Wang+CY&cauthor_id=23663078), [Jaya Ramaprasad](https://pubmed.ncbi.nlm.nih.gov/?size=100&term=Ramaprasad+J&cauthor_id=23663078), [Anne McTiernan](https://pubmed.ncbi.nlm.nih.gov/?size=100&term=McTiernan+A&cauthor_id=23663078). Predictors of adherence to a 26-week viniyoga intervention among post-treatment breast cancer survivors**.** J Altern Complement Med. 2013 Sep;19(9):751-8. doi: 10.1089/acm.2012.0118. PMID: **23663078.** PMCID: PMC3768230. |
|  | Janet Douglass, Maarten Immink, Neil Piller, Shahid Ullah. Yoga for women with breast cancer-related lymphoedema: a preliminary 6-month study. Journal of Lymphoedema, 2012, Vol 7, No 2. |
|  | Jeanne M. Ferrante, Aaron Lulla, Julie D. Williamson, Katie A. Devine, Pamela Ohman-Strickland, Elisa V. Bandera. Patterns of Fitbit Use and Activity Levels Among African American Breast Cancer Survivors During an eHealth Weight Loss Randomized Controlled Trial. Am J Health Promot. 2022 Jan;36(1):94-105. doi: 10.1177/08901171211036700. PMID: **34344171.** PMCID: PMC8918046. |
|  | Mary Lou Galantino, Krupali Desai, Laurie Greene, Angela DeMichele, Carrie Tompkins Stricker, Jun James Mao. Impact of Yoga on Functional Outcomes in Breast Cancer Survivors With Aromatase Inhibitor–Associated Arthralgias. Integr Cancer Ther. 2012 Dec;11(4):313-20. doi: 10.1177/1534735411413270. PMID: **21733988.** |
|  | Devesh Oberoi, [Andrew McLennan](https://pubmed.ncbi.nlm.nih.gov/?size=100&term=McLennan+A&cauthor_id=33902334), [Katherine-Ann Piedalue](https://pubmed.ncbi.nlm.nih.gov/?size=100&term=Piedalue+KA&cauthor_id=33902334), [Peter M Wayne](https://pubmed.ncbi.nlm.nih.gov/?size=100&term=Wayne+PM&cauthor_id=33902334), [Jennifer M Jones](https://pubmed.ncbi.nlm.nih.gov/?size=100&term=Jones+JM&cauthor_id=33902334), [Linda E Carlson](https://pubmed.ncbi.nlm.nih.gov/?size=100&term=Carlson+LE&cauthor_id=33902334). Factors Influencing Preference for Intervention in a Comparative Effectiveness Trial of Mindfulness-Based Cancer Recovery and Tai Chi/ Qigong in Cancer Survivors. J Altern Complement Med. 2021 May;27(5):423-433. doi: 10.1089/acm.2020.0400. PMID: **33902334.** |
|  | Leonessa Boing, Maria de Fátima Marcelina Baptista, Gustavo Soares Pereira, Fabiana Flores Sperandio, Jéssica Moratelli, Allana Alexandre Cardoso, Adriano, Ferreti Borgatto, Adriana Coutinho de Azevedo Guimarães. Benefits of belly dance on quality of life, fatigue, and depressive symptoms in women with breast cancer – A pilot study of a non-randomised clinical trial. J Bodyw Mov Ther. 2018 Apr;22(2):460-466. doi: 10.1016/j.jbmt.2017.10.003. PMID: **29861250.** |
|  | Lim JM. The effects of home-based exercise education on quality of life index (SF-30) for breast cancer resection patients. Korea J Sports Sci. 2012;21:1139-47. |
|  | Jo Fields, Alison Richardson, Jane Hopkinson, Deborah Fenlon. Nordic Walking as an Exercise Intervention to Reduce Pain in Women with Aromatase Inhibitor Associated Arthralgia: A Feasibility Study. Journal of Pain and Symptom Management. 2016 Oct;52(4):548-559. doi: 10.1016/j.jpainsymman.2016.03.010. PMID: **27265816.** |
|  | Rebecca N. Latka, Marty Alvarez-Reeves, Lisa Cadmus, Melinda L. Irwin. Adherence to a randomized controlled trial of aerobic exercise in breast cancer survivors: the Yale exercise and survivorship study. J Cancer Surviv (2009) 3:148–157. DOI 10.1007/s11764-009-0088-z. PMID: **19626443.** |
|  | Scherezade K. Mama, Jaejoon Song, Alexis Ortiz, Maribel Tirado-Gomez, Cristina Palacios, Daniel C. Hughes6 and Karen Basen-Engquist. Longitudinal social cognitive influences on physical activity and sedentary time in Hispanic breast cancer survivors. Psychooncology. 2017 Feb;26(2):214-221. doi: 10.1002/pon.4026. PMID: **26602701.** PMCID: PMC4879102. |
| **Reason 2. Study type: protocol** | |
|  | L. Boing, T. Bem Fretta, M. Souza Vieira, G. Soares Pereira, J. Moratelli, F. F. Sperandio, A. Bergmann, F. Baptista, M. Dias, A. Coutinho de Azevedo Guimarães. Pilates and dance to patients with breast cancer undergoing treatment: study protocol for a randomized clinical trial – MoveMama study. Trials (2020). 21 (1): 35. doi: 10.1186/s13063-019-3874-6. PMID: **31910872.** PMCID: PMC6947954. |
|  | Siobhan M. Phillipsa, Linda M. Collinsb, Frank J. Penedoc, Kerry S. Courneyad, Whitney Welcha, Alison Cottrella, Gillian R. Lloyda, Kara Gavina, David Cellab, Ronald T. Ackermanne, Juned Siddiquea, Bonnie Springa. Optimization of a technology-supported physical activity intervention for breast cancer survivors: Fit2Thrive study protocol. Contemp Clin Trials. 2018 Mar;66:9-19. doi: 10.1016/j.cct.2018.01.001. PMID: **29330081.** PMCID: PMC5828903. |
|  | Siobhan Phillips; Payton Solk; Whitney Welch; Lisa Auster-Gussman; Marilyn Lu; Erin Cullather; Emily Torre; Madelyn Whitaker; Emily Izenman; Jennifer La; Jungwha Lee; Bonnie Spring; William Gradishar. A Technology-Based Physical Activity Intervention for Patients With Metastatic Breast Cancer (Fit2ThriveMB): Protocol for a Randomized Controlled Trial. JMIR Res Protoc. 2021 Apr 23;10(4):e24254. doi: 10.2196/24254. PMID: **33890857.** PMCID: PMC8105756. |
|  | Supa Pudkasam, Meron Pitcher, Melanie Fisher, Anne O’Connor, Nanthaphan Chinlumprasert, Lily Stojanovska, Remco Polman, Vasso Apostolopoulos. The PAPHIO study protocol: a randomised controlled trial with a 2 x 2 crossover design of physical activity adherence, psychological health and immunological outcomes in breast cancer survivors. BMC Public Health (2020) 20:696 https://doi.org/10.1186/s12889-020-08827-x. |
|  | Luiz Augusto Riani Costa, Raphael F. Barreto, Sarah Milani Moraes de Leandrini, Aline Rachel Bezerra Gurgel, Gabriel Toledo de Sales, Vanessa Azevedo Voltarelli, Gilberto de Castro Jr., Sally A. M. Fenton, James E. Turner, Christian Klausener, Lucas Melo Neves, Carlos Ugrinowitsch, Jose Carlos Farah, Cláudia Lúcia de Moraes Forjaz, Christina May Moran Brito, Patricia Chakur Brum. The influence of a supervised group exercise intervention combined with active lifestyle recommendations on breast cancer survivors’ health, physical functioning, and quality of life indices: study protocol for a randomized and controlled trial. Riani Costa et al. Trials (2021) 22:934 https://doi.org/10.1186/s13063-021-05843-z. PMID: **34922621.** PMCID: PMC8684206. |
|  | [Marina Touillaud](https://pubmed.ncbi.nlm.nih.gov/?size=100&term=Touillaud+M&cauthor_id=34518245), [Bautista Fournier](https://pubmed.ncbi.nlm.nih.gov/?size=100&term=Fournier+B&cauthor_id=34518245), [Olivia Perol](https://pubmed.ncbi.nlm.nih.gov/?size=100&term=P%C3%A9rol+O&cauthor_id=34518245), [Lidia Delrieu](https://pubmed.ncbi.nlm.nih.gov/?size=100&term=Delrieu+L&cauthor_id=34518245), [Aurelia Maire](https://pubmed.ncbi.nlm.nih.gov/?size=100&term=Maire+A&cauthor_id=34518245), [Elodie Belladame](https://pubmed.ncbi.nlm.nih.gov/?size=100&term=Belladame+E&cauthor_id=34518245), [david perol](https://pubmed.ncbi.nlm.nih.gov/?size=100&term=P%C3%A9rol+D&cauthor_id=34518245), [Lionel Perrier](https://pubmed.ncbi.nlm.nih.gov/?size=100&term=Perrier+L&cauthor_id=34518245), [María Preau](https://pubmed.ncbi.nlm.nih.gov/?size=100&term=Preau+M&cauthor_id=34518245), [tanguy leroy](https://pubmed.ncbi.nlm.nih.gov/?size=100&term=Leroy+T&cauthor_id=34518245), [Jean-Baptiste Fassier](https://pubmed.ncbi.nlm.nih.gov/?size=100&term=Fassier+JB&cauthor_id=34518245), [Florie Fillol](https://pubmed.ncbi.nlm.nih.gov/?size=100&term=Fillol+F&cauthor_id=34518245), [Sébastien Pascual](https://pubmed.ncbi.nlm.nih.gov/?size=100&term=Pascal+S&cauthor_id=34518245), [Thierry Durand](https://pubmed.ncbi.nlm.nih.gov/?size=100&term=Durand+T&cauthor_id=34518245), [Beatrice Fervers](https://pubmed.ncbi.nlm.nih.gov/?size=100&term=Fervers+B&cauthor_id=34518245). Connected device and therapeutic patient education to promote physical activity among women with localised breast cancer (DISCO trial): protocol for a multicentre 2×2 factorial randomised controlled trial. BMJ Open. 2021 Sep 13;11(9):e045448. doi: 10.1136/bmjopen-2020-045448. PMID: **34518245.** PMCID: PMC8438826. |
|  | Kajal Gokal, Fehmidah Munir, Deborah Wallis, Samreen Ahmed, Ion Boiangiu, Kiran Kancherla. Can physical activity help to maintain cognitive functioning and psychosocial well-being among breast cancer patients treated with chemotherapy? A randomised controlled trial: study protocol. BMC Public Health. 2015 Apr 23;15:414. doi: 10.1186/s12889-015-1751-0. PMID: **25903195.** PMCID: PMC4446147. |
|  | Annette Loudon, Tony Barnett, Neil Piller, Maarten A Immink, Denis Visentin, Andrew D Williams. The effect of yoga on women with secondary arm lymphoedema from breast cancer treatment. BMC Complementary and Alternative Medicine 2012, 12:66. |
| **Reason 3. Congress** | |
|  | Sandi Hayes, Diana Battistutta, Elizabeth Eakin. Evaluating telephone versus face-to-face modes of exercise intervention delivery to women during and following treatment for breast cancer. Queensland University of Technology, Kelvin Grove, Qld, Australia. |
|  | Gokal K, Munir F, Wallis D, Ahmed S, Boiangiu I, Kancherla K. Physical activity intervention for cognitive & emotional functioning in breast cancer patients receiving chemotherapy. Psycho-Oncology 2013;22:254. |
| **Reason 4. Thesis** | |
|  | Song HS. The effects of shoulder joint mobilization and scapular complex exercise on body function and psycho-social factors in after patient with breast cancer surgery [dissertation]. Daejeon: Daejeon Univ.; 2016. |
|  | Kim JH. Effects of combined exercise on upper extremity function, pain and self-efficacy in survivors with breast cancer after surgery [master’s thesis]. Busan: Silla Univ.; 2017. |
|  | Do JH. Effects of resistance exercises and complex decongestive therapy on arm function and muscular strength in breast cancer related lymph edema [dissertation]. Ulsan: Ulsan Univ.; 2017. |
| **Reason 5. Different types of cancer were included** | |
|  | Aasha I. Hoogland, Suzanne C. Lechner, Brian D. Gonzalez, Brent J. Small, Dinorah M. Tyson, Yasmin Asvat, Anna Barata, Maria F. Gomez, Yvelise Rodriguez, Heather S. L. Jim, Michael H. Antoni, Paul B. Jacobsen, Cathy D. Meade. Efficacy of a Spanish-Language Self-Administered Stress Management Training Intervention for Latinas Undergoing Chemotherapy. Psychooncology. 2018 Apr;27(4):1305-1311. doi: 10.1002/pon.4673. PMID: **29462503.** PMCID: PMC5895519. |
|  | Caroline S. Kampshoff, Willem van Mechelen, Goof Schep, Marten R. Nijziel, Lenja Witlox, Lisa Bosman, Mai J. M. Chinapaw, Johannes Brug, Laurien M. Buffart. Participation in and adherence to physical exercise after completion of primary cancer treatment. Int J Behav Nutr Phys Act. 2016 Sep 9;13(1):100. doi: 10.1186/s12966-016-0425-3. PMID: **27612561.** PMCID: PMC5016937 |
|  | Sarah Kozey Keadle, Leah Meuter, Suzanne Phelan, Siobhan M. Phillips. Charity‑based incentives motivate young adult cancer survivors to increase physical activity: a pilot randomized clinical trial. J Behav Med. 2021 Oct;44(5):682-693. doi: 10.1007/s10865-021-00218-w. PMID: **33825070** |
|  | Hoang Tran, Cheng Lin, Fang Yu, Angie Frederick, Molly Mieras, Lorena Baccaglini. A multicenter study on the relative effectiveness of a 12-week physical training program for adults with an oncologic diagnosis. Support Care Cancer. 2016 Sep;24(9):3705-13. doi: 10.1007/s00520-016-3194-2. PMID: **27037811.** |
|  | Vicki Tsianakas, Jenny Harris, Emma Ream, Mieke Van Hemelrijck, Arnie Purushotham, Lorelei Mucci, James S A Green, Jacquetta Fewster, Jo Armes. CanWalk: a feasibility study with embedded randomised controlled trial pilot of a walking intervention for people with recurrent or metastatic cancer. BMJ Open. 2017 Feb 15;7(2):e013719. doi: 10.1136/bmjopen-2016-013719. PMID: **28202500.** PMCID: PMC5318561. |
|  | Nadine Ungar, Monika Sieverding, Gerdi Weidner, Cornelia M. Ulrich, Joachim Wiskemann. A self-regulation-based intervention to increase physical activity in cancer patients. Psychol Health Med. 2016;21(2):163-75. doi: 10.1080/13548506.2015.1081255. PMID: **26367803.** |
|  | Carmina G. Valle, Deborah F. Tate, Deborah K. Mayer, Marlyn Allicock, Jianwen Cai. A randomized trial of a Facebook-based physical activity intervention for young adult cancer survivors. J Cancer Surviv. 2013 Sep;7(3):355-68. doi: 10.1007/s11764-013-0279-5. PMID: **23532799.** PMCID: PMC3737370. |
|  | Carmina G. Valle, Deborah F. Tate, Deborah K. Mayer, Marlyn Allicock, Jianwen Cai. Exploring Mediators of Physical Activity in Young Adult Cancer Survivors: Evidence from a Randomized Trial of a Facebook-Based Physical Activity Intervention. J Adolesc Young Adult Oncol. 2015 Mar;4(1):26-33. doi: 10.1089/jayao.2014.0034. PMID: **25852972.** PMCID: PMC4365511. |
|  | Hsiao‐Lan Wang, Kristine A. Donovan, Sahana Rajasekhara, Tapan Padhya, Harleah G. Buck, Laura Szalacha, J. Morris Chang, Jaelyn D. Brown, Barbara Smith. The pre‐efficacy phase testing for PAfitME™—A behavioral physical activityintervention tomanagemoderate and severe symptoms among advanced stage cancer patients. Res Nurs Health. 2021 Feb;44(1):238-249. doi: 10.1002/nur.22099. PMID: **33373078.** |
|  | J. Webb a, C. Fife-Schaw, J. Ogden. A randomised control trial and cost-consequence analysis to examine the effects of a print-based intervention supported by internet tools on the physical activity of UK cancer survivors. Public Health. 2019 Jun;171:106-115. doi: 10.1016/j.puhe.2019.04.006. PMID: **31121557.** |
| **Reason 6. Multimodal interventions** | |
|  | Roger T., Anderson, Gretchen G., Kimmick, Thomas P., McCoy, J. Hopkins, E. Levine, G. Miller, P. Ribisl, S. L. Mihalko. A randomized trial of exercise on well-being and function following breast cancer surgery: the RESTORE trial. J Cancer Surviv (2012) 6:172–181. DOI 10.1007/s11764-011-0208-4. PMID: **22160629.** PMCID: PMC3900279. |
|  | Maryam Changizi, Leila Ghahremani, Niloofar Ahmadloo, Mohammad Hossein Kaveh. The Patient Health Engagement Model in Cancer Management: Effect of Physical Activity, Distress Management, and Social Support Intervention to Improve the Quality of Life in Breast Cancer Patients. International Journal of Breast Cancer.  2022 Apr 30;2022:1944852. doi: 10.1155/2022/1944852. PMID: **35535128.** PMCID: PMC9078844 |
|  | Leonard, S M, Milos, A, ML, Galantino, Schmid, P,Botis, S, Dagan, C, SM, Leonard, Milos, A. Preliminary Effectiveness Study of a Community-Based Wellness Coaching for Cancer Survivors Program. Rehabilitation Oncology. 28(1):19-25, 2010. https://doi.org/10.1177/15598276221076040. |
|  | Linda M. Ercoli, Steven A. Castellon, Aimee M. Hunter, Lorna Kwan, Barbara A. Kahn-Mills, Paul A. Cernin, Andrew F. Leuchter, Patricia A. Ganz. Assessment of the feasibility of a rehabilitation intervention program for breast cancer survivors with cognitive complaints. Brain Imaging and Behavior (2013) 7 (4):543–553. DOI 10.1007/s11682-013-9237-0. PMID: **23955490** |
|  | Mary Jane Esplen, Jiahui Wong, Ellen Warner, and Brenda Toner. Restoring Body Image After Cancer (ReBIC): Results of a Randomized Controlled Trial. Journal of Clinical Oncology. 2018 Mar 10;36(8):749-756. PMID: **29356610.** DOI: 10.1200/JCO.2017.74.8244 |
|  | Edgar Gonzalez-Hernandez, Rocio Romero, Daniel Campos, Diana Burichka, Rebeca Diego-Pedro, Rosa Baños, Lobsang Tenzin Negi, Ausiàs Cebolla. Cognitively-Based Compassion Training (CBCT) in Breast Cancer Survivors: A Randomized Clinical Trial Study. Integr Cancer Ther. 2018 Sep;17(3):684-696. doi: 10.1177/1534735418772095. PMID: **29681185.** PMCID: PMC6142100. |
|  | Rachel Hirschey, Gretchen Kimmick, Marilyn Hockenberry, Ryan Shaw, Wei Pan, Christina Page, Isaac Lipkus. A Randomized Phase II Trial of MOVING ON: An Intervention to Increase Exercise Outcome Expectations among Breast Cancer Survivors. Psychooncology. 2018 Oct;27(10):2450-2457. doi: 10.1002/pon.4849. PMID: **30071146.** PMCID: PMC6684254 |
|  | Mario Lozano-Lozano, Lydia Martín-Martín, Noelia Galiano-Castillo, Francisco Álvarez-Salvago, Irene Cantarero-Villanueva, Carolina Fernández-Lao, Carmen Sánchez-Salado, Manuel Arroyo-Morales. Integral strategy to supportive care in breast cancer survivors through occupational therapy and a m-health system: design of a randomized clinical trial. BMC Med Inform Decis Mak. 2016 Nov 25;16(1):150. doi: 10.1186/s12911-016-0394-0. PMID: **27887610.** PMCID: PMC5124301. |
|  | Daniel Oluwafemi Odebiyi, Adebusola Temitayo Aborowa, Oluwaleke Ganiyu Sokunbi, Happiness Anulika Aweto, Aderemi Tajudeen Ajekigbe. Effects of exercise and oedema massage on fatigue level and quality of life of female breast cancer patients. European Journal of Physiotherapy. Volume 16, 2014 - [Issue 4](https://www.tandfonline.com/toc/iejp20/16/4). https://doi.org/10.3109/21679169.2014.959048 |
|  | Karl Reif, Ulrike de Vries b, Franz Petermann, Stefan Görres. A patient education program is effective in reducing cancer-related fatigue: A multi-centre randomised two-group waiting-list controlled intervention trial. Eur J Oncol Nurs. 2013 Apr;17(2):204-13. doi: 10.1016/j.ejon.2012.07.002. PMID: **22898654.** |
|  | Laura Q. Rogers, Stephen Markwell, Patricia Hopkins-Price, Sandy Vicari, Kerry S. Courneya, Karen Hoelzer, Steven Verhulst. Reduced Barriers Mediated Physical Activity Maintenance Among Breast Cancer Survivors. J Sport Exerc Psychol. 2011 April ; 33(2): 235–254. PMID: **21558582.** PMCID: PMC3145412. |
|  | Laura Q. Rogers, Kerry S. Courneya, Phillip M. Anton, Patricia Hopkins-Price, Steven Verhulst, Randall S. Robbs, Sandra K. Vicari, Edward McAuley. Social Cognitive Constructs Did Not Mediate the BEAT Cancer Intervention Effects on Objective Physical Activity Behavior Based on Multivariable Path Analysis. Ann Behav Med. 2017 Apr;51(2):321-326. doi: 10.1007/s12160-016-9840-6. PMID: **27752993.** PMCID: PMC5373935. |
|  | Stacie Scruggs, Scherezade K. Mama, Cindy L. Carmack, Tommy Douglas, Pamela Diamond, Karen Basen-Engquist. Randomized Trial of a Lifestyle Physical Activity Intervention for Breast Cancer Survivors: Effects on Transtheoretical Model Variables. Health Promot Pract. 2018 Jan;19(1):134-144. doi: 10.1177/1524839917709781. PMID: **28627254.** |
|  | Tine Kova, Miha Kova. Impact of Relaxation Training According to Yoga in Daily Life  System on Self-Esteem After Breast Cancer Surgery. J Altern Complement Med. 2011 Dec;17(12):1157-64. doi: 10.1089/acm.2010.0653. PMID: **22106845.** |
|  | Thais R. S. Paulo, Fabricio E. Rossi, Juliana Viezel, Giuliano T. Tosello, Sylvia C. Seidinger, Regina R. Simões, Ruffo de Freitas, Ismael F. Freitas. The impact of an exercise program on quality of life in older breast cancer survivors undergoing aromatase inhibitor therapy: a randomized controlled trial. Health Qual Life Outcomes. 2019 Jan 18;17(1):17. doi: 10.1186/s12955-019-1090-4. PMID: **30658629.** PMCID: PMC6339353. |
|  | Nanette Mutrie, Anna Campbell, Sarah Barry, Kate Hefferon, Alex McConnachie, Diana Ritchie, Sian Tovey. Five-year follow-up of participants in a randomised controlled trial showing benefits fromexercise for breast cancer survivors during adjuvant treatment. Are there lasting effects? J Cancer Surviv. 2012 Dec;6(4):420-30. doi: 10.1007/s11764-012-0233-y. PMID: **22836201.** PMCID: PMC3505536. |
|  | Bernardine M. Pinto, Shira I. Dunsiger, Madison M. Kindred, Sheryl Mitchell. Peer mentoring for physical activity adoption and maintenance among breast cancer survivors: moderators of physical activity outcomes. J Cancer Surviv. 2022 Jan 7. doi: 10.1007/s11764-021-01162-z. PMID: **34994945.** |
|  | Elisabeth Kenne Sarenmalm, Lena B Mårtensson, Stig B Holmberg, Bengt A Andersson, Anders Odén, Ingrid Bergh. Mindfulness based stress reduction study design of a longitudinal randomized controlled complementary intervention in women with breast cancer. *BMC Complementary and Alternative Medicine* volume 13, Article number: 248 (2013). http://www.biomedcentral.com/1472-6882/13/248. |
|  | Rebecca Crane-Okada, Holly Kiger, Fred Sugerman, Gwen C. Uman, Shauna L. Shapiro, Wendy Wyman-McGinty, Nancy L. R. Anderson. Mindful Movement Program for Older Breast Cancer Survivors. Cancer Nurs. 2012 Jul-Aug;35(4):E1-13. doi: 10.1097/NCC.0b013e3182280f73. PMID: **22705939.** |
|  | Soheila Rahmani, Siavash Talepasand. The effect of group mindfulness - based stress reduction program and conscious yoga on the fatigue severity and global and specific life quality in women with breast cancer. Med J Islam Repub Iran. 2015 Feb 8;29:175. PMID: **26034728.** PMCID: PMC4431452. |
|  | Sheila Pintado, Sandra Andrade. Randomized controlled trial of mindfulness program to enhance body image in patients with breast cancer. [European Journal of Integrative Medicine](https://www.sciencedirect.com/journal/european-journal-of-integrative-medicine) [Volume 12](https://www.sciencedirect.com/journal/european-journal-of-integrative-medicine/vol/12/suppl/C), June 2017, Pages 147-152. https://doi.org/10.1016/j.eujim.2017.05.009. |
|  | Madison M. Kindred, Bernardine M. Pinto, Shira I. Dunsiger. Mediators of physical activity adoption and maintenance among breast cancer survivors. J Behav Med. 2020 Aug;43(4):605-613. doi: 10.1007/s10865-019-00085-6. PMID: **31377976.** |
|  | Bernardine M. Pinto, Georita M. Frierson, Carolyn Rabin, Joseph J. Trunzo, Bess H. Marcus. Home-Based Physical Activity Intervention for Breast Cancer Patients. J Clin Oncol. 2005 May 20;23(15):3577-87. doi: 10.1200/JCO.2005.03.080. PMID: **15908668.** |
|  | Spence RR, Sandler CX, Singh B, Tanner J, Pyke C, Eakin E, Vagenas D, Hayes SC. A Randomised, Comparative, Effectiveness Trial Evaluating Low- versus High-Level Supervision of an Exercise Intervention for Women with Breast Cancer: The SAFE Trial. Cancers (Basel). 2022 Mar 16;14(6):1528. doi: 10.3390/cancers14061528. PMID: 35326679; PMCID: PMC8946819. |
| **Reason 7. Results do not show study variables of interest** | |
|  | Freerk T. Baumann, Oliver Bieck, Max Oberste, Rafaela Kuhn, Joachim Schmitt, Steffen Wentrock, Eva Zopf, Wilhelm Bloch, Klaus Schüle, Monika Reuss-Borst. Sustainable impact of an individualized exercise program on physical activity level and fatigue syndrome on breast cancer patients in two German rehabilitation centers. Support Care Cancer 2017 25 (4). DOI 10.1007/s00520-016-3490-x. PMID: **27942857.** |
|  | Stephen J. Carter, Gary R. Hunter, Edward McAuley, Kerry S. Courneya, Philip M. Anton, Laura Q. Rogers. Lower rate-pressure product during submaximal walking: a link to fatigue improvement following a physical activity intervention among breast cancer survivors. J Cancer Surviv. 2016 Oct;10(5):927-34. PMID: **27061740.** PMCID: PMC5018414. DOI: 10.1007/s11764-016-0539-2 |
|  | Hsiang-Ping Huang, Fur-Hsing Wen, Jen-Chen Tsai, Yung-Chang Lin, Shiow-Ching Shun, Hsien-Kun Chang, Jong-Shyan Wang, Sui-Whi Jane, Min-Chi Chen, Mei-Ling Chen. Adherence to prescribed exercise time and intensity declines as the exercise program proceeds: findings from women under treatment for breast cancer. Support Care Cancer. 2015 Jul;23(7):2061-71. doi: 10.1007/s00520-014-2567-7. PMID: **25527243.** |
|  | Marize Ibrahim, Thierry Muanza, Nadia Smirnow, Warren Sateren, Beatrice Fournier, Petr Kavan, Michael Palumbo, Richard Dalfen, Mary-Ann Dalzell. Time course of upper limb function and return-to-work post-radiotherapy in young adults with breast cancer: a pilot randomized control trial on effects of targeted exercise program. J Cancer Surviv. 2017 Dec;11(6):791-799. doi: 10.1007/s11764-017-0617-0. PMID: **28470507** |
|  | Melinda L. Irwin, Lisa Cadmus, Marty Alvarez-Reeves, Mary O’Neil, Eileen Mierzejewski, Rebecca Latka, Herbert Yu, Loretta DiPietro, Beth Jones, M. Tish Knobf, Gina G. Chung, Susan T. Mayne. Recruiting and Retaining Breast Cancer Survivors into a Randomized Controlled Exercise Trial. Cancer. 2008 Jun 1;112(11 Suppl):2593-606. doi: 10.1002/cncr.23446. PMID: **18428192.** PMCID: PMC5450159. |
|  | Ian M. Lahart, Amtul R. Carmichael, Alan M. Nevill, George D. Kitas, George S. Metsios. The effects of a home-based physical activity intervention on cardiorespiratory fitness in breast cancer survivors; a randomised controlled trial. J Sports Sci. 2018 May;36(10):1077-1086. doi: 10.1080/02640414.2017.1356025. PMID: **28745131.** |
|  | Charles E. Matthews, Sara Wilcox, Cara L. Hanby, Chery Der Ananian, Sue P. Heiney, Tebeb Gebretsadik, Ayumi Shintani. Evaluation of a 12-week home-based walking intervention for breast cancer survivors. Support Care Cancer (2007) 15: 203–211 DOI 10.1007/s00520-006-0122-x. PMID: **17001492.** |
|  | Camille E. Short, Erica L. James, Afaf Girgis, Mario I D’Souza Ronald C. Plotnikoff. Main outcomes of the Move More for Life Trial: a randomised controlled trial examining the effects of tailored-print and targeted-print materials for promoting physical activity among post-treatment breast cancer survivors. Psychooncology. 2015 Jul;24(7):771-8. doi: 10.1002/pon.3639. PMID: **25060288.** |
|  | Raheleh Soleimani, Ahmad Ali Eslami, Mohammad Almasian, Akbar Hassanzadeh, Negar Ra’isi Dehkordi, Noushin Parsa Gohar, and Fariborz Mokarian Rajabi. The Effect of Self-Efficacy and Outcome Expectations Training on the Enhancement of Physical Activities Among Women Suffering From Breast Cancer: An Evidence-Based Intervention. Iran Red Crescent Med J. 2016 December; 18(12):e28081. doi: 10.5812/ircmj.28081. |
|  | Jeff Vallance; Ronald C. Plotnikoff; Kristina H. Karvinen; John R. Mackey; Kerry S. Courneya. Understanding Physical Activity Maintenance in Breast Cancer Survivors. Am J Health Behav. 2010 Mar-Apr;34(2):225-36. doi: 10.5993/ajhb.34.2.10. PMID: **19814602.** |
|  | Kerri M. Winters-Stone, Monica Laudermilk, Kaitlin Woo, Justin C. Brown, Kathryn H. Schmitz. Influence of weight training on skeletal health of breast cancer survivors with or at risk for breast cancer-related lymphedema. J Cancer Surviv. 2014 Jun;8(2):260-8. doi: 10.1007/s11764-013-0337-z. PMID: **24390808.** PMCID: PMC4448695. |
|  | Kerri M. Winters-Stone, Britta Torgrimson-Ojerio, Nathan F. Dieckmann, Sydnee Stoyles, Zahi Mitri, Shiuh-Wen Luoh. A randomized-controlled trial comparing supervised aerobic training to resistance training followed by unsupervised exercise on physical functioning in older breast cancer survivors. J Geriatr Oncol. 2022 Mar;13(2):152-160. doi: 10.1016/j.jgo.2021.08.003. PMID: **34426142.** PMCID: **PMC9003120.** |
|  | [Heather J Leach](https://pubmed.ncbi.nlm.nih.gov/?size=100&term=Leach+HJ&cauthor_id=31319398), [Katie B Potter](https://pubmed.ncbi.nlm.nih.gov/?size=100&term=Potter+KB&cauthor_id=31319398), [Mary C Hidde](https://pubmed.ncbi.nlm.nih.gov/?size=100&term=Hidde+MC&cauthor_id=31319398). A Group Dynamics-Based Exercise Intervention to Improve Physical Activity Maintenance in Breast Cancer Survivors**.** J Phys Act Health. 2019 Sep 1;16(9):785-791. doi: 10.1123/jpah.2018-0667. PMID: **31319398.** |
|  | [Marjorie K McClure](https://pubmed.ncbi.nlm.nih.gov/?size=100&term=McClure+MK&cauthor_id=20131565), [Richard J McClure](https://pubmed.ncbi.nlm.nih.gov/?size=100&term=McClure+RJ&cauthor_id=20131565), [Richard Day](https://pubmed.ncbi.nlm.nih.gov/?size=100&term=Day+R&cauthor_id=20131565), [Adam M Brufsky](https://pubmed.ncbi.nlm.nih.gov/?size=100&term=Brufsky+AM&cauthor_id=20131565). Randomized controlled trial of the Breast Cancer Recovery Program for women with breast cancer-related lymphedema. Am J Occup Ther. 2010 Jan-Feb;64(1):59-72. doi: 10.5014/ajot.64.1.59. PMID: **20131565.** |
|  | Jessica McNeil, Mina Fahim, Chelsea R. Stone, Rachel O’Reilly, Kerry S. Courneya, Christine M. Friedenreich. Adherence to a lower versus higher intensity physical activity intervention in the Breast Cancer & Physical Activity Level (BC-PAL) Trial. J Cancer Surviv. 2022 Apr;16(2):353-365. doi: 10.1007/s11764-021-01030-w. PMID: **33754246.** |
|  | V Mock, [K H Dow](https://pubmed.ncbi.nlm.nih.gov/?size=100&term=Dow+KH&cauthor_id=9243585), [C J Meares](https://pubmed.ncbi.nlm.nih.gov/?size=100&term=Meares+CJ&cauthor_id=9243585), [P M Grimm](https://pubmed.ncbi.nlm.nih.gov/?size=100&term=Grimm+PM&cauthor_id=9243585), [J A Dienemann](https://pubmed.ncbi.nlm.nih.gov/?size=100&term=Dienemann+JA&cauthor_id=9243585), [M E Haisfield-Wolfe](https://pubmed.ncbi.nlm.nih.gov/?size=100&term=Haisfield-Wolfe+ME&cauthor_id=9243585), [W Quitasol](https://pubmed.ncbi.nlm.nih.gov/?size=100&term=Quitasol+W&cauthor_id=9243585), [S Mitchell](https://pubmed.ncbi.nlm.nih.gov/?size=100&term=Mitchell+S&cauthor_id=9243585), [A Chakravarthy](https://pubmed.ncbi.nlm.nih.gov/?size=100&term=Chakravarthy+A&cauthor_id=9243585), [I Gage](https://pubmed.ncbi.nlm.nih.gov/?size=100&term=Gage+I&cauthor_id=9243585). Effects of exercise on fatigue, physical functioning, and emotional distress during radiation therapy for breast cancer. Oncol Nurs Forum. 1997 Jul;24(6):991-1000. PMID: **9243585.** |
|  | Mary Pickett, Victoria Mock, Mary E. Ropka, Lane Cameron, Meghan Coleman, Laura Podewils. Adherence to moderate-intensity exercise during breast cancer therapy. Cancer Pract. 2002 Nov-Dec;10(6):284-92. doi: 10.1046/j.1523-5394.2002.106006.x. PMID: **12406050.** |
|  | [Hanneke Poort](https://pubmed.ncbi.nlm.nih.gov/?size=100&term=Poort+H&cauthor_id=34435804), [Fabiola Müller](https://pubmed.ncbi.nlm.nih.gov/?size=100&term=M%C3%BCller+F&cauthor_id=34435804), [Gijs Bleijenberg](https://pubmed.ncbi.nlm.nih.gov/?size=100&term=Bleijenberg+G&cauthor_id=34435804), [Stans A H H V M Verhagen](https://pubmed.ncbi.nlm.nih.gov/?size=100&term=Verhagen+SAHHVM&cauthor_id=34435804), [Mathilde G E Verdam](https://pubmed.ncbi.nlm.nih.gov/?size=100&term=Verdam+MGE&cauthor_id=34435804), [Pythia T Nieuwkerk](https://pubmed.ncbi.nlm.nih.gov/?size=100&term=Nieuwkerk+PT&cauthor_id=34435804), [Hans Knoop](https://pubmed.ncbi.nlm.nih.gov/?size=100&term=Knoop+H&cauthor_id=34435804). Condition or cognition? Mechanism of change in fatigue in a randomized controlled trial of graded exercise therapy or cognitive behavior therapy for severe fatigue in patients with advanced cancer. J Consult Clin Psychol. 2021 Sep;89(9):731-741. doi: 10.1037/ccp0000670. PMID: **34435804.** |
|  | [Kamli Prakash](https://pubmed.ncbi.nlm.nih.gov/?size=100&term=Prakash+K&cauthor_id=33311874), [Sunil K Saini](https://pubmed.ncbi.nlm.nih.gov/?size=100&term=Saini+SK&cauthor_id=33311874), [Sanchita Pugazhendi](https://pubmed.ncbi.nlm.nih.gov/?size=100&term=Pugazhendi+S&cauthor_id=33311874). Effectiveness of Yoga on Quality of Life of Breast Cancer Patients Undergoing Chemotherapy: A Randomized Clinical Controlled Study. Indian J Palliat Care. 2020 Jul-Sep;26(3):323-331. doi: 10.4103/IJPC.IJPC_192_19. PMID: **33311874.** PMCID: PMC7725186. |
|  | [Sheri J Hartman](https://pubmed.ncbi.nlm.nih.gov/?size=100&term=Hartman+SJ&cauthor_id=31605514), [Lauren S Weiner](https://pubmed.ncbi.nlm.nih.gov/?size=100&term=Weiner+LS&cauthor_id=31605514), [Sandahl H Nelson](https://pubmed.ncbi.nlm.nih.gov/?size=100&term=Nelson+SH&cauthor_id=31605514), [Loki Natarajan](https://pubmed.ncbi.nlm.nih.gov/?size=100&term=Natarajan+L&cauthor_id=31605514), [Ruth E Patterson](https://pubmed.ncbi.nlm.nih.gov/?size=100&term=Patterson+RE&cauthor_id=31605514), [Barton W Palmer](https://pubmed.ncbi.nlm.nih.gov/?size=100&term=Palmer+BW&cauthor_id=31605514), [Barbara A Parker](https://pubmed.ncbi.nlm.nih.gov/?size=100&term=Parker+BA&cauthor_id=31605514), [Dorothy D Sears](https://pubmed.ncbi.nlm.nih.gov/?size=100&term=Sears+DD&cauthor_id=31605514). Mediators of a Physical Activity Intervention on Cognition in Breast Cancer Survivors: Evidence From a Randomized Controlled Trial**.** JMIR Cancer. 2019 Oct 11;5(2):e13150. doi: 10.2196/13150. PMID: **31605514.** PMCID: PMC6914286. |
|  | CE Short, A Rebar, EL James, MJ Duncan, KS Courneya, RC Plotnikoff, R Crutzen, C Vandelanotte. How do different delivery schedules of tailored web-based physical activity advice for breast cancer survivors influence intervention use and efficacy? J Cancer Surviv. 2017 Feb;11(1):80-91. doi: 10.1007/s11764-016-0565-0. PMID: **27498099.** |
|  | A. H. Uludağ, F. Ardahan, H. Bozcuk. The effects of regular physical exercise on the values of the physical properties and body compositions of breast cancer patients in remission. Pamukkale Journal of Sport Sciences . 2018, Vol. 9 Issue 3, p31-39. 9p. |
|  | Niina Vähäaho, Liisa Hakamies-Blomqvist, Carl Blomqvist, Pirkko-Liisa Kellokumpu-Lehtinen, Riikka Huovinen, Tiina Saarto, Christian Hakulinen. Sense of Coherence as Predictor of Quality of Life in Early Breast Cancer Patients. Anticancer Res. 2021 Oct;41(10):5045-5052. doi: 10.21873/anticanres.15319. PMID: **34593453.** |
|  | Hee J. Yoo, Se H. Ahn, Sung B. Kim, Woo K. Kim, Oh S. Han. Efficacy of progressive muscle relaxation training and guided imagery in reducing chemotherapy side effects in patients with breast cancer and in improving their quality of life. Support Care Cancer (2005) 13: 826–833. DOI 10.1007/s00520-005-0806-7. PMID: **15856335.** |
|  | Hon Keung Yuen, David Sword. Home-based exercise to alleviate fatigue and improve functional capacity among breast cancer survivors. J Allied Health. 2007 Winter;36(4):e257-75. PMID: **19759996.** |
|  | K L Campbell, J W Y Kam, S E Neil-Sztramko, T Liu Ambrose, T C Handy, H J Lim, S Hayden, L Hsu, A A Kirkham, C C Gotay, D C McKenzie, L A Boyd. Effect of aerobic exercise on cancer-associated cognitive impairment: A proof-of-concept RCT. Psychooncology. 2018 Jan;27(1):53-60. doi: 10.1002/pon.4370. PMID: **28075038.** |
|  | Helen M. Milne, Karen E. Wallman, Sandy Gordon, Kerry S. Courneya. Effects of a combined aerobic and resistance exercise program in breast cancer survivors: a randomized controlled trial. Breast Cancer Res Treat. 2008 Mar;108(2):279-88. doi: 10.1007/s10549-007-9602-z. PMID: **17530428.** |
|  | Heather J. Leach, Kelley R. Covington, Corrine Voss, Kelli A. LeBreton, Samantha M. Harden, Steven R. Schuster. Effect of Group Dynamics–Based Exercise Versus Personal Training in Breast Cancer Survivors. Oncol Nurs Forum. 2019 Mar 1;46(2):185-197. doi: 10.1188/19.ONF.185-197. PMID: **30767964.** |
|  | Abdou Y. Omorou, Didier Peiffert, Christine Rotonda, Aurélie Van Hoye, Edem Allado, Oriane Hily, Margaux Temperelli, Bruno Chenuel, Dominique Hornus-Dragne, Mathias Poussel. Adapted Fencing for Patients With Invasive Breast Cancer: The RIPOSTE Pilot Randomized Controlled Trial. Front Sports Act Living. 2022 Mar 29;4:786852. doi: 10.3389/fspor.2022.786852. PMID: **35425895.** PMCID: PMC9002110. |
|  | Frank M. Perna, Lynette Craft, Karen M. Freund, Gary Skrinar, Michael Stone, Lisa Kachnic, Carolyn Youren, Tracy A. Battaglia. The Effect of a Cognitive Behavioral Exercise Intervention on Clinical Depression in a Multiethnic Sample of Women With Breast Cancer: A Randomized Controlled Trial. International Journal of Sport and Exercise Psychology, 8:1, 36-47. https://doi.org/10.1080/1612197X.2010.9671932. |
|  | V Mock, M Pickett, M E Ropka, E Muscari Lin, K J Stewart, V A Rhodes, R McDaniel, P M Grimm, S Krumm, R McCorkle. Fatigue and quality of life outcomes of exercise during cancer treatment. Cancer Pract. 2001 May-Jun;9(3):119-27. doi: 10.1046/j.1523-5394.2001.009003119.x. PMID: **11879296.** |
|  | Pinto BM, Rabin C, Dunsiger S. Home-based exercise among cancer survivors: adherence and its predictors. Psychooncology. 2009 Apr;18(4):369-76. doi: 10.1002/pon.1465. PMID: 19242921; PMCID: PMC2958525. |
|  | Lenja Witlox, Miranda J. Velthuis, Jennifer H. Boer, Charlotte N. Steins Bisschop, Elsken van der Wall, Wout J. T. M. van der Meulen, Carin D. Schro¨ der, Petra H. M. Peeters, Anne M. May. Attendance and compliance with an exercise program during localized breast cancer treatment in a randomized controlled trial: The PACT study. PLoS One. 2019 May 8;14(5):e0215517. doi: 10.1371/journal.pone.0215517. PMID: **31067223.** PMCID: PMC6505930. |
|  | Debra Reis, M. Eileen Walsh, Stacey Young-McCaughan, Tisha Jones. Effects of Nia Exercise in Women Receiving Radiation Therapy for Breast Cancer. Oncol Nurs Forum. 2013 Sep;40(5):E374-81. doi: 10.1188/13.ONF.E374-E381. PMID: **23989030.** |
|  | Anouk E Hiensch, Kate A Bolam, Sara Mijwel, Anne M May, Yvonne Wengström. Sense of coherence and its relationship to participation, cancer-related fatigue, symptom burden, and quality of life in women with breast cancer participating in the OptiTrain exercise trial. Support Care Cancer. 2020 Nov;28(11):5371-5379. doi: 10.1007/s00520-020-05378-0. PMID: **32140973.** PMCID: PMC7546973. |
|  | Hülya Özlem Şener, Mehtap Malkoç, Gülbin Ergin, Didem Karadibak, Tuğba Yavuzşen. Effects of Clinical Pilates Exercises on Patients Developing Lymphedema after Breast Cancer Treatment: A Randomized Clinical Trial. J Breast Health 2017; 13: 16-22. DOI: 10.5152/tjbh.2016.3136. PMID: **28331763.** PMCID: PMC5351459. |
|  | Helen M. Milne, Karen E. Wallman, Sandy Gordon, Kerry S. Courneya. Impact of a Combined Resistance and Aerobic Exercise Program on Motivational Variables in Breast Cancer Survivors: A Randomized Controlled Trial. ann. behav. med. (2008) 36:158–166. DOI 10.1007/s12160-008-9059-2. PMID: **18795388.** |
|  | Renate M. Winkels, Kathleen M. Sturgeon, Michael J. Kallan, Lorraine T. Dean, Zi Zhang, Margaret Evangelisti, Justin C. Brown, David B. Sarwer, Andrea B. Troxel, Crystal Denlinger, Monica Laudermilk, Anna Fornash, Angela DeMichele, Lewis A. Chodosh, Kathryn H. Schmitz. The women in steady exercise research (WISER) survivor trial: The innovative transdisciplinary design of a randomized controlled trial of exercise and weight-loss interventions among breast cancer survivors with lymphedema. Contemp Clin Trials. 2017 Oct;61:63-72. doi: 10.1016/j.cct.2017.07.017. PMID: **28739540.** PMCID: PMC5817634. |
|  | Raghavendra Mohan Rao, Nagarathna Raghuram, Nagendra, MR Usharani, KS Gopinath, Ravi B Diwakar, Shekar Patil, Ramesh S Bilimagga, Nalini Rao. Effects of an integrated Yoga Program on Self-reported Depression Scores in Breast Cancer Patients Undergoing Conventional Treatment: A Randomized Controlled Trial. Indian J Palliat Care. 2015 May-Aug;21(2):174-81. doi: 10.4103/0973-1075.156486. PMID: **26009671.** PMCID: PMC4441179. |
|  | Anna K. Koch, Sybille Rabsilber, Romy Lauche, Sherko K¨ummel, Gustav Dobos, Jost Langhorst, Holger Cramer. The effects of yoga and self-esteem on menopausal symptoms and quality of life in breast cancer survivors—A secondary analysis of a randomized controlled trial. Maturitas. 2017 Nov;105:95-99. doi: 10.1016/j.maturitas.2017.05.008. PMID: **28551083.** |
|  | Simona Micheletti, Patrizia Serra, Anna Tesei, Irene Azzali, Chiara Arienti, Valentina Ancarani, Stefania Corelli, Antonino Romeo, Giovanni Martinelli. Effects of yoga practice on physiological distress, fatigue and QOL in patients affected by breast cancer undergoing adjuvant radiotherapy. Tech Innov Patient Support Radiat Oncol. 2022 Sep 23;24:32-39. doi: 10.1016/j.tipsro.2022.09.005. PMID: **36176568.** PMCID: PMC9513264. |
|  | Raghavendra Mohan Rao, Nagaratna Raghuram, Hongasandra Ramarao Nagendra, Gopinath S Kodaganur, Ramesh S Bilimagga, HP Shashidhara, Ravi B Diwakar, Shekhar Patil, Nalini Rao. Effects of a Yoga Program on Mood States, Quality of Life, and Toxicity in Breast Cancer Patients Receiving Conventional Treatment: A Randomized Controlled Trial. Indian J Palliat Care. 2017 Jul-Sep;23(3):237-246. doi: 10.4103/IJPC.IJPC_92_17. PMID: **28827925.** PMCID: PMC5545947. |
|  | Laura S. Porter, James W. Carson, Maren Olsen, Kimberly M. Carson, Linda Sanders, Lee Jones, Kelly Westbrook, Francis J. Keefe. Feasibility of a mindful yoga program for women with metastatic breast cancer: results of a randomized pilot study. Support Care Cancer. 2019 Nov;27(11):4307-4316. doi: 10.1007/s00520-019-04710-7. PMID: **30877596.** PMCID: PMC6745290. |
|  | Lee JS, Lee BK, Kim TS. Effects of 4 wks classical decongestive physiotherapy and combined therapeutic exercises on edema, upper limb function and quality of life for middle-aged women with upper lymphedema after breast cancer surgery. J Sport Leis Stud. 2012;50:879-89. |
|  | Seo JY, Choi JD. The effects of sensory motor training using ball exercise on shoulder functions and quality of life in breast cancer women after mastectomy. J Korean Soc Phys Med. 2016;11:147-56. |
|  | Cornette T, Vincent F, Mandigout S, et al. Effects of home-based exercise training on VO2 in breast cancer patients under adjuvant or neoadjuvant chemotherapy (SAPA): a randomized controlled trial. Eur J Phys Rehabil Med. 2016;52(2):223–232. |
|  | Bernardine M. Pinto, Matthew M. Clark, Nancy C. Maruyama, Susan I. Feder. Psychological and fitness changes associated with exercise participation among women with breast cancer. Psycho-Oncology 12: 118–126 (2003). DOI: 10.1002/pon.618. PMID: **12619144.** |
|  | Helen M. Milne, Karen E. Wallman, Sandy Gordon, Kerry S. Courneya. Effects of a combined aerobic and resistance exercise program in breast cancer survivors: a randomized controlled trial. Breast Cancer Res Treat (2008) 108:279–288. DOI 10.1007/s10549-007-9602-z. PMID: **17530428.** |
|  | Amanda J. Daley, Helen Crank, Nanette Mutrie, John M. Saxton, Robert Coleman. Determinants of adherence to exercise in women treated for breast cancer. European Journal of Oncology Nursing (2007) 11, 392–399. doi: 10.1016/j.ejon.2007.03.001. PMID: **17524796.** |
|  | Thorsten Schmidt, Burkhard Weisser, Juliane Dürkop, Walter Jonat, Marion Van Mackelenbergh, Christoph Röcken, Christoph Mundhenke. Comparing Endurance and Resistance Training with Standard Care during Chemotherapy for Patients with Primary Breast Cancer. *Anticancer Research 35: 5623-5630 (2015).* PMID: **26408735.** |
|  | Kerry S. Courneya, John R. Mackey, Gordon J. Bell, Lee W. Jones, Catherine J. Field, Adrian S. Fairey. Randomized Controlled Trial of Exercise Training in Postmenopausal Breast Cancer Survivors: Cardiopulmonary and Quality of Life Outcomes. J Clin Oncol. 2003 May 1;21(9):1660-8. doi: 10.1200/JCO.2003.04.093. PMID: **12721239.** |
|  | Amanda J. Daley, Helen Crank, John M. Saxton, Nanette Mutrie, Robert Coleman, Andrea Roalfe. Randomized Trial of Exercise Therapy in Women Treated for Breast Cancer. J Clin Oncol. 2007 May 1;25(13):1713-21. doi: 10.1200/JCO.2006.09.5083. PMID: **17470863.** |
|  | Karen M. Mustian, Oxana G. Palesh, Stephanie A. Flecksteiner. Tai Chi Chuan for Breast Cancer Survivors. Med Sport Sci. 2008 ; 52: 209–217. doi:10.1159/000134301. PMID: **18487900.** PMCID: PMC3927648. |
|  | Kathryn H. Schmitz, Andrea B. Troxel, Andrea Cheville, Lorita L. Grant, Cathy J. Bryan, Cynthia Gross, Leslie A. Lytle, Rehana L. Ahmed. Physical Activity and Lymphedema (The PAL Trial): Assessing the safety of progressive strength training in breast cancer survivors. Contemp Clin Trials. 2009 May ; 30(3): 233–245. doi:10.1016/j.cct.2009.01.001. PMID: **19171204.** PMCID: PMC2730488. |
| **Reason 8. Intervention does not fit with our physical exercise definition** | |
|  | Courneya KS, McKenzie DC, Gelmon K, Mackey JR, Reid RD, Yasui Y, Friedenreich CM, Forbes CC, Trinh L, Jespersen D, Cook D, Proulx C, Wooding E, Dolan LB, Segal RJ. A multicenter randomized trial of the effects of exercise dose and type on psychosocial distress in breast cancer patients undergoing chemotherapy. Cancer Epidemiol Biomarkers Prev. 2014 May;23(5):857-64. doi: 10.1158/1055-9965.EPI-13-1163. Epub 2014 Mar 5. PMID: 24599578. |
|  | Sandel SL, Judge JO, Landry N, Faria L, Ouellette R, Majczak M. Dance and movement program improves quality-of-life measures in breast cancer survivors. Cancer Nurs. 2005 Jul-Aug;28(4):301-9. doi: 10.1097/00002820-200507000-00011. PMID: 16046894. |
|  | Gokal K, Wallis D, Ahmed S, Boiangiu I, Kancherla K, Munir F. Effects of a self-managed home-based walking intervention on psychosocial health outcomes for breast cancer patients receiving chemotherapy: a randomised controlled trial. Support Care Cancer. 2016 Mar;24(3):1139-66. doi: 10.1007/s00520-015-2884-5. Epub 2015 Aug 15. PMID: 26275768. |

**Table S3**. Description of the included studies.

| **Author(s), year, country, RoB** | **Sample size**  **(mean age, SD)**  ***Undergoing primary adjuvant treatment**** | ***Exercise prescription***  ***(Un)Supervised, individual/group*** | **Comparison** | **Outcomes and assessment points** | **Main findings** |
| --- | --- | --- | --- | --- | --- |
| ***Boing et al., 2023***  ***[51]***  Brasil | 50 participants (55 ± 10 years old)  EG = 25 (54 ± 10 years old)  CG = 24 (57 ± 11 years old)  *No adjuvant treatment* | EG:  16-week of Mat Pilates  48 sessions  (60 minutes, 3 days/week)  *Supervised, group* | CG: educational sessions about stretching, lymphedema prevention and self-esteem and body image  3 sessions (N/A) | Self-esteem: Rosenberg Self-Esteem Scale  Body Image: Body Image After Breast Cancer Questionnaire (Body Stigma subscale)  T_0_: baseline, T_1_: 16 weeks, T_2_: 24 weeks, T_3_: 52 weeks | All groups showed a time effect in self-esteem from baseline to 52 weeks (p=0.05) |
| ***Cadmus et al., 2009***  ***[56]***  USA | 75 participants (40 - 75 years old)  EG = 37 (56.5 ± 9.5 years old)  CG = 38 (55.1 ± 7.7 years old)  *No adjuvant treatment* | EG: 26-week aerobic exercises (60-80% HR_max_)  130 sessions  (30 minutes, 5 days/week)  *Supervised, individual* | CG: Usual routine activities | Self-esteem: Rosenberg Self-Esteem Scale  T_0_: baseline, T_1_: 26 weeks | There were not statistically significant differences between EG CG (p > 0.05) |
| ***Courneya et al., 2003***  ***[47]***  USA | 53 participants (59 ± 6; 50 – 69 years old)  EG = 24 (59 ± 5 years old)  CG = 28 (58 ± 6 years old)  *No adjuvant treatment* | EG: 15-week aerobic exercises (70- 75% VO_2max_)  45 sessions  (weeks 1-3: 15 min increased by 5 minutes/3 weeks until 35 minutes, 3 days/week)  *Supervised, individual* | CG: Usual routine activities | Self-esteem: Rosenberg Self-Esteem Scale  T_0_: baseline, T_1_: 15 weeks | There were statistically significant differences in favour of EG (p = 0.01). Moreover, mean self-esteem was lower in the CG at T_1_ |
| ***Courneya et al., 2007a***  ***[34]***  USA  ***Courneya et al., 2007b***  ***[35]***  USA | 242 participants (49.2 ± no specified; 25 - 78 years old)  EG 1 = 82 (49.5 ± no specified; 25 - 76 years old)  EG 2 = 78 (49.0 ± no specified; 30 - 75 years old)  CG = 82 (49 ± no specified; 26 - 78 years old)  *Chemotherapy* | EG 1: Aerobic exercises (ergometer, treadmill or elliptical 60-80% VO2_max_)  During chemotherapy administration (15-45 min. progressively, 3 days/week)  EG 2: Resistance exercises (9 exercises, 60-70% 1RM)  During chemotherapy administration (8-12 repetitions, increased10%, 3 days/week)  *Supervised, individual* | CG: Usual routine activities | Self-esteem: Rosenberg Self-Esteem Scale  T_0_: baseline, T_1_: 24 weeks | There were statistically significant difference sbetween CG and EG2 in favour of EG (p<0.05)  EG1 showed a trend toward higher self-esteem compared wit CG but it was not significant |
| ***Do et al., 2015***  ***[52]***  Korea | 62 participants  EG = 32 (47.1 ± 8.5 years old)  CG = 30 (48.3 ± 8.2 years old)  *No adjuvant treatment* | EG: 4-week multimodal exercises (aerobic exercise: treadmill, bicycle, stepper machine 60-75% VO2_max;_ strengthening exercises 60-80% 1RM; core stability)  20 sessions (80 min: 40 min aerobic exercises, 20 min resistance exercises; 20 min warn up-cold down, 5 days/week)  *Supervised, individual* | CG: Usual routine activities | Body image: EORTC QLQ-BR23 (Body Image subscale)    T_0_: baseline; T_1_: 4 weeks | There were no statistically significant differences between EG and CG (p > 0.05) |
| ***Haines et al., 2010***  ***[44]***  Australia | 89 participants  EG = 46 (55.9 ± 10.5 years old)  CG = 43 (54.2 ± 11.5 years old)  *Chemotherapy, radiation therapy* | EG: Six-month multimodal exercises program (strength, balance, shoulder mobility and cardiovascular endurance)  Nº sessions: N/A (20 min walking, 10-15 rep mobility, 5-15 rep/2 sets 8 exercises)  *Unsupervised (home-based, DVD), individual* | CG: Sham flexibility and relaxation program  Nº sessions, min: N/A | Body image: EORTC QLQ-BR23 (Body Image subscale)  T_0_: baseline; T_1_: 12 weeks; T_2_: 24 weeks | There were no statistically significant differences between EG and CG (all p > 0.05) |
| ***Landry et al., 2018***  ***[46]***  France | 34 participants  EG1 = 17 (51 ± 2 years old)  EG2 = 17 (54 ± 3 years old)  *Chemotherapy, radiation therapy* | EG: 12-week multimodal exercises (muscle strengthening, balance, and flexibility)  12 sessions (60 min, 1day/week)  *N/A* | CG: Usual routine activities | Self-esteem: Rosenberg Self-Esteem Scale  T_0_: baseline; T_1_: 6 weeks; T_2_: 12 weeks | There were statistical differences between groups in favour of EG at T_1_ and T_2_ (p < 0.05) |
| ***Leite et al., 2021***  ***[50]***  Brazil | 74 participants (55 ± 10 years old)  EG = 25 (53 ± 8 years old)  CG = 24 (58 ± 11 years old)  *No adjuvant treatment* | EG: 16-week Mat Pilates  48 sessions (60 min, 3 days/week)  *Supervised, group* | CG: Usual routine activities + educational sessions about stretching, lymphedema prevention and self-esteem and body image  3 sessions (N/A) | Self-esteem: Rosenberg Self-Esteem Scale  T_0_: baseline; T_1_: 16 weeks | There were no statically significant differences (all p > 0.05) |
| ***Mehnert et al., 2011***  ***[48]***  Germany | 63 participants (51.88 ± 8.46 years old)  EG = 35 (53.03 ± 7.40 years old)  CG = 28 (50.64 ± 9.44 years old)  *No adjuvant treatment* | EG: 10- week aerobic exercises (gymnastics, movement games and relaxation; walking, and jogging, 60% VO_2max_)  20 sessions (90 min, 2 days/week)  *Supervised, group* | CG: Usual routine exercises | Body image: Body Image Questionnaire  T_0_: baseline; T_1_: 10 weeks | There were no statistically significant differences between EG and CG (p > 0.05) |
| ***Musanti et al., 2011***  ***[55]***  USA | 55 participants (50.5 ± 7.5 years old)  EG1 = 12 (51 ± 5.5 years old)  EG2 = 17 (52 ± 8.9 years old)  EG3 = 13 (48 ± 6.7 years old)  CG = 13 (52 ±7.9 years old)  *No adjuvant treatment* | EG1 = 12-week aerobic exercises (walking, 40-65% up to 85% HR_max_)  36 sessions (15-30 min, 3 days/week)  EG2 = 12-week endurance exercises  36 sessions (10-12 rep, 10 exercises, 3 days/week)  EG3 = 12- week multimodal exercises (EG1+EG2 intervention)  48-60 sessions aerobic exercise (15-30 min, 4-5 days/week)  24 sessions endurance exercises (10-12 rep, 10 exercises, 2 days/week)  All groups performed flexibility exercises (warm-up routine)  *Unsupervised (home-based), individual* | CG: Flexibility exercises (warm-up routine)  *Unsupervised (home-based), individual* | Self-esteem: Rosenberg Self-Esteem Scale  Body image:  Physical Self-perception Profile (Attractive Body domain)  T_0_: baseline; T_1_: 12 weeks | There were no statistically significant differences between groups  EG2 improved body image significantly (p<0.01) |
| ***Mustian et al., 2004***  ***[54]***  USA | 31 participants (52±9 years old)  EG: 17 (N/A) years old  CG: 14 (N/A) years old  *No adjuvant treatment* | EG: 12-week Tai Chi Chuan Exercise  36 sessions (60 min, 3 days/week)  *Supervised, group* | CG: Psychosocial Support Therapy  36 sessions (60 min, 3 days/week)  *Supervised, group* | Self-esteem: Rosenberg Self-Esteem Scale  T_0_: baseline; T_1_: 6 weeks; T_2_: 12 weeks | There were statistical differences between groups in favour of EG at T_2_ (p = 0.01), not in T_1_ (p = 0.40) |
| ***Pinto et al., 2003***  ***[30]***  USA | 24 participants (52.5± 6.8 years old)  EG: 12  CG: 12  *No adjuvant treatment* | EG: 12-week Aerobic Exercise (60-70% HR_max_) + upper limb endurance the last 4 weeks (light weights (1-5lbs)  36 sessions (50 min, 3 days/eek)  *Unsupervised, individual* | CG: Usual routine exercises | Self-esteem: Body Esteem Scale (Physical Condition, Physical Attractiveness, Weight concern subscales)  T_0_: baseline; T_1_: 12 weeks | Changes in the Physical Condition and Weight Concern subscales among the EG were significantly higher than in the CG (p=0.03) |
| ***Saarto et al., 2012***  ***[49]***  Finland | 573 participants (35-68 years old)  EG: 302, 52.3 (N/A) years old  CG: 271, 52,4 (N/A) years old  *No adjuvant treatment* | EG: 12- month aerobic exercises (75-up to 90% HR_max_)  156 sessions (60 min 3 days/week)  *1 day/week supervised, group*  *2 days/week unsupervised, individual* | CG: Usual routine exercises | Body image: EORTC QLQ-BR23 (Body Image subscale)  T_0_: baseline; T_1_: 12 months | There were not significant differences between groups |
| ***Salchow et al., 2021***  ***[23]***  Germany | 51 participants  EG: 31 (54.23±7.84 years old)  CG: 20 (51.52±8.41 years old)  *No adjuvant treatment* | EG: 24-week holistic Kyusho Jitsu  48 sessions (90 min, 2 days/week)  *Supervised, group* | CG: Usual routine exercises | Self-efficacy: German SWE (Selbst-Wirksamkeits-Erwartung) questionnaire  T_0_: baseline; T_1_: 24 weeks | There were not significant differences between groups |
| ***Segar et al., 1998***  ***[33]***  USA | 24 participants (48.9±7.6 years old)  EG: 16 (47.5 ±7.1 years old)  CG: 8 (51.8±8.1 years old)  *No adjuvant treatment* | EG: 10-week aerobic exercises (60% HR_max_)  40 sessions (30 min, 4 days/week)  *Unsupervised, individual* | CG: Usual routine exercises | Self-esteem: Rosenberg Self-Esteem Scale  T_0_: baseline; T_1_: 10 weeks; T_2_: 22 weeks | There were not significant differences between groups |
| ***Speck et al., 2010***  ***[53]***  USA | 215 participants (56.5, 36-80)  EG: 104 (N/A)  CG: 111 (N/A)  *No adjuvant treatment* | EG: 53-week multimodal exercises (aerobic, endurance, stretching)  106 sessions (90 min, 2days/week)  *First weeks supervised, 40 weeks unsupervised, group* | CG: Usual routine exercises | Body image: Body Image and Relationships Scale (Appearance and Sexuality subscale)  T_0_: baseline; T_1_: 12 months | There were statistical differences between groups in favour of EG (p: 0.004) |
| ***Steindorf et al., 2014***  ***[45]***  Germany | 160 participants (55.8 ± 9.1 years old)  EG: 80 (55.2 ± 9.5 years old)  CG: 80 (56.4±8.7 years old)  *Radiation therapy* | EG: 12-week resistance exercises (eight different machine-based exercises; 3 sets, 8–12 repetitions at 60%–80% of 1RM)  24 sessions (60 min, 2 days/week)  *Supervised, group* | CG: 12-week muscle relaxation  24 sessions (60 min, 2 days/week)  *Supervised, group* | Body image: EORTC QLQ-BR23 (Body Image subscale)  T_0_: baseline; T_1_: 13 weeks | There were not significant differences between groups |
| ***Wang et al., 2021***  ***[31]***  USA | 60 participants (56 ± 10 years old)  EG: 31 (55.6±8.2 years old)  CG: 29 (56 ±11,5 years old)  *No adjuvant treatment* | EG: 16-week endurance exercises  *3 supervised individual sessions during the intervention, rest sessions unsupervised without an imposed frequency or duration* | CG: Usual routine exercises | Self-efficacy: Self-Efficacy and Physical Activity Scale  T_0_: baseline; T_1_: 16 weeks | Significant differences were observed in favour of the EG (p=0.047) |
| ***Winter-Stone et al., 2017***  ***[32]***  USA | 95 participants  EG: 50 (54.7±10.3 years old)  CG: 45 (60.8±10.5 years old)  *25% of participants receiving chemotherapy or radiation therapy* | EG: 8-week instructional yoga + oncologist’s general recommendations to exercise  A mean of 16 sessions (30 min, 2 days/week)  *Unsupervised (DVD), individual* | CG: Oncologist’s general recommendations to exercise | Self-efficacy for exercise: Self-Efficacy Questionnaire  T_0_: baseline; T_1_: 4 weeks; T_2_: 8 weeks | There were not significant differences between groups |

*We do not consider hormonal treatment.

**Abbreviations: CG** = Control group; ***EG*** = experimental group; **EORTC QLQ-BR23** = European Organization for Research and Treatment of Cancer Quality of Life Questionnaire; **HRmax**= maximal heart rate; **min**= minutes; **rep=** repetition; **RM**= maximal repetition; **VO2max=** maximal oxygen consumption.

**Table S4.** TIDier Checklist items.

| **Items checklist of TIDier** | Boing et al, 2023  Brazil | Cadmus et al, 2009  USA | Courneya et al, 2003  USA | Courneya et al, 2007a,b    USA | Do et al, 2015 Korea | Haines et al, 2010 Australia | Landry et al, 2017  France | Leite et al, 2021 USA | Mehnert et al, 2011 Germany | Musanti et al, 2011 USA | Mustian et al, 2004  USA |
| --- | --- | --- | --- | --- | --- | --- | --- | --- | --- | --- | --- |
| 1. **Brief name** | ✓ | ✓ | ✓ | ✓ | ✓ | ✓ | ✓ | ✓ | ✓ | ✓ | ✓ |
| 1. **Why** | ✓ | ✓ | ✓ | ✓ | ✓ | ✓ | 🗶 | ✓ | ✓ | ✓ | ✓ |
| 1. **What: *materials*** | ✓ | ✓ | ✓ | ✓ | ✓ | ✓ | 🗶 | ✓ | 🗶 | 🗶 | ✓ |
| 1. **What: *procedures*** | ✓ | 🗶 | 🗶 | ✓ | ✓ | ✓ | ✓ | ✓ | ✓ | ✓ | ✓ |
| 1. **Who provided** | ✓ | ✓ | ✓ | ✓ | ✓ | ✓ | 🗶 | 🗶 | ✓ | 🗶 | 🗶 |
| 1. **How** | ✓ | ✓ | ✓ | ✓ | ✓ | ✓ | 🗶 | ✓ | ✓ | ✓ | ✓ |
| 1. **Where** | ✓ | ✓ | 🗶 | 🗶 | ✓ | ✓ | 🗶 | ✓ | 🗶 | 🗶 | 🗶 |
| 1. **When and how much** | ✓ | ✓ | ✓ | ✓ | ✓ | 🗶 | ✓ | ✓ | ✓ | ✓ | ✓ |
| 1. **Tailoring** | ✓ | 🗶 | ✓ | ✓ | ✓ | ✓ | 🗶 | ✓ | ✓ | ✓ | 🗶 |
| 1. **Modifications*** | 🗶 | 🗶 | 🗶 | 🗶 | 🗶 | 🗶 | 🗶 | 🗶 | 🗶 | 🗶 | 🗶 |
| 1. **How well: *planed*** | 🗶 | ✓ | 🗶 | 🗶 | 🗶 | 🗶 | 🗶 | 🗶 | ✓ | 🗶 | 🗶 |
| 1. **How well: *actual**** | 🗶 | ✓ | 🗶 | 🗶 | 🗶 | 🗶 | 🗶 | ✓ | 🗶 | 🗶 | 🗶 |

| **Items checklist of TIDier** | Pinto et al, 2003  USA | Saarto et al, 2012 Finland | Salchow et al, 2021; Germany | Segar et al, 1998; USA | Speck et al, 2010; USA | Steinford et al, 2014; Germany | Wang et al, 2021; USA | Winter-Stone et al, 2021; USA |
| --- | --- | --- | --- | --- | --- | --- | --- | --- |
| 1. **Brief name** | ✓ | ✓ | ✓ | ✓ | ✓ | ✓ | ✓ | ✓ |
| 1. **Why** | ✓ | ✓ | ✓ | ✓ | ✓ | ✓ | ✓ | ✓ |
| 1. **What: *materials*** | 🗶 | ✓ | 🗶 | 🗶 | 🗶 | ✓ | ✓ | ✓ |
| 1. **What: *procedures*** | ✓ | ✓ | ✓ | ✓ | ✓ | ✓ | ✓ | ✓ |
| 1. **Who provided** | ✓ | ✓ | 🗶 | 🗶 | ✓ | ✓ | ✓ | ✓ |
| 1. **How** | ✓ | ✓ | ✓ | ✓ | ✓ | ✓ | ✓ | ✓ |
| 1. **Where** | 🗶 | ✓ | ✓ | 🗶 | ✓ | ✓ | ✓ | 🗶 |
| 1. **When and how much** | ✓ | ✓ | ✓ | ✓ | ✓ | ✓ | ✓ | 🗶 |
| 1. **Tailoring** | ✓ | ✓ | ✓ | ✓ | ✓ | ✓ | ✓ | 🗶 |
| 1. **Modifications*** | 🗶 | 🗶 | 🗶 | ✓ | 🗶 | ✓ | 🗶 | 🗶 |
| 1. **How well: *planed*** | 🗶 | 🗶 | 🗶 | 🗶 | 🗶 | 🗶 | 🗶 | 🗶 |
| 1. **How well: *actual**** | 🗶 | 🗶 | 🗶 | 🗶 | 🗶 | 🗶 | 🗶 | 🗶 |

**Table S5.** Certainty of evidence (GRADE) in breast cancer participants that received exercise interventions.

| Summary of findings | | |  |  | | Certainty in evidence based on the GRADE approach | | | | | |
| --- | --- | --- | --- | --- | --- | --- | --- | --- | --- | --- | --- |
| Outcome | Studies, n (k) | Participants (N/S) | Risk of bias | | Inconsistency | | Indirectness | Imprecision | Publication bias | Level of evidence | Importance |
| Body image | 8 (10) | 1171/1216 | Very Serious^1^  (-2) | | No | | Very Serious^2^  (-2) | No | Yes^3^  (-1) | Very Low | Critical |
| Self-esteem | 6 (9) | 405/608 | Serious^4^  (-1) | | No | | Very Serious^2^  (-2) | No | No | Very Low | Critical |

Note: GRADE = Grading of Recommendations Assessment, Development and Evaluation. N: sample of primary studies in meta-analysis not considering the multi-arm; n: number of studies; k: number of arms of studies included in meta-analysis; S: complete sample included in the meta-analysis considering all multi-arms. The results of GRADE were based on the meta-analyses findings.

1. Downgrade two level due to most information is from RCTs with high or some concerns of bias with potential limitations that are likely to lower confidence in the estimate of effect.

2. Downgrade two levels due to large heterogeneity between experimental interventions and measurement of outcomes.

3. Downgrade one level due to the presence of publication bias without reasonable explanation.

4. Downgrade one level due to most information is from RCTs with high or some concerns of bias with potential limitations that are likely to lower confidence in the estimate of effect.

**Figure S6.** Sensitivity analysis for self-perceived body image outcome.


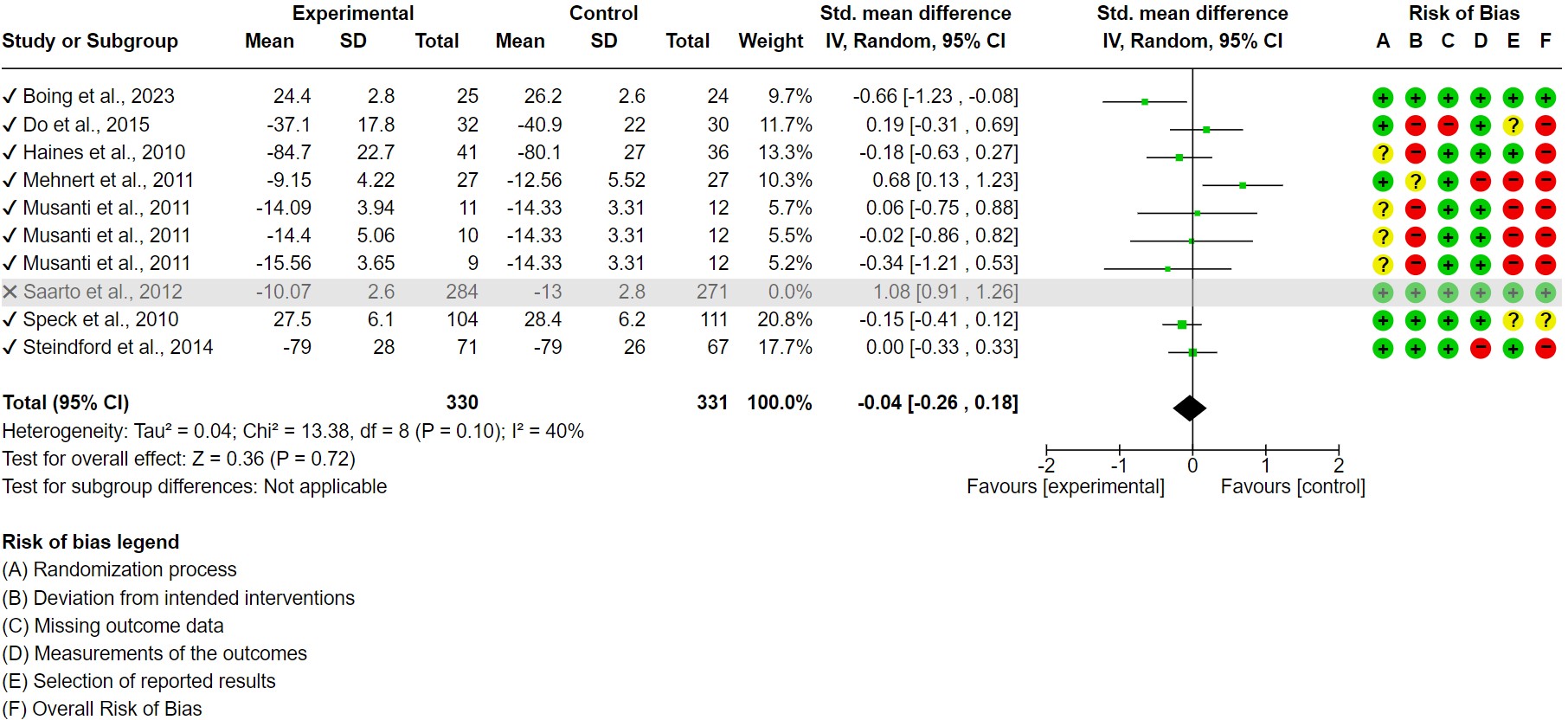


**Table S7.** Subgroup meta-analyses for self-perceived body image.

| Subgroup (number of arms of studies meta-analyzed) | SMD (95% CI, p-value) | I^2^ | Test for between subgroup differences  Chi^2^ (p-value)* |
| --- | --- | --- | --- |
| **Body image (10)** |  |  |  |
| *Type of exercise*  Endurance/aerobic (3)  Resistance/strength (3)  Multimodal (4) | 0.70 (0.14 to 1.27)  -0.27 (-0.72 to 0.17)  -0.09 (-0.29 to 0.12) | 74%  48%  0% | **7.92 (p=0.02)*** |
| *Exercise interventions in group*  Yes (5)  No (5) | 0.21(-0.47 to 0.88)  -0.04 (-0.32 to 0.23) | 95%  0% | 0.44 (p=0.51) |
| *Supervised sessions*  Yes (6)  No (4) | 0.20 (-0.38 to 0.79)  0.14(-0.47 to 0.19) | 94%  0% | 1.01 (p=0.32) |
| *Risk of bias (RoB-2)*  High (7)  Low (2)  Some concerns (1) | 0.07 (-0.15 to 0.30)  0.12 (-1.35 to 1.60)  -0.15 (-0.41 to 0.12) | 18%  96%  NA | 1.54 (P=0.46) |
| *p-value <0.05 indicates between subgroups heterogeneity; NA: not applicable | | | |

**Figure S8.** Subgroup analysis for self-perceived body image (type of exercise).


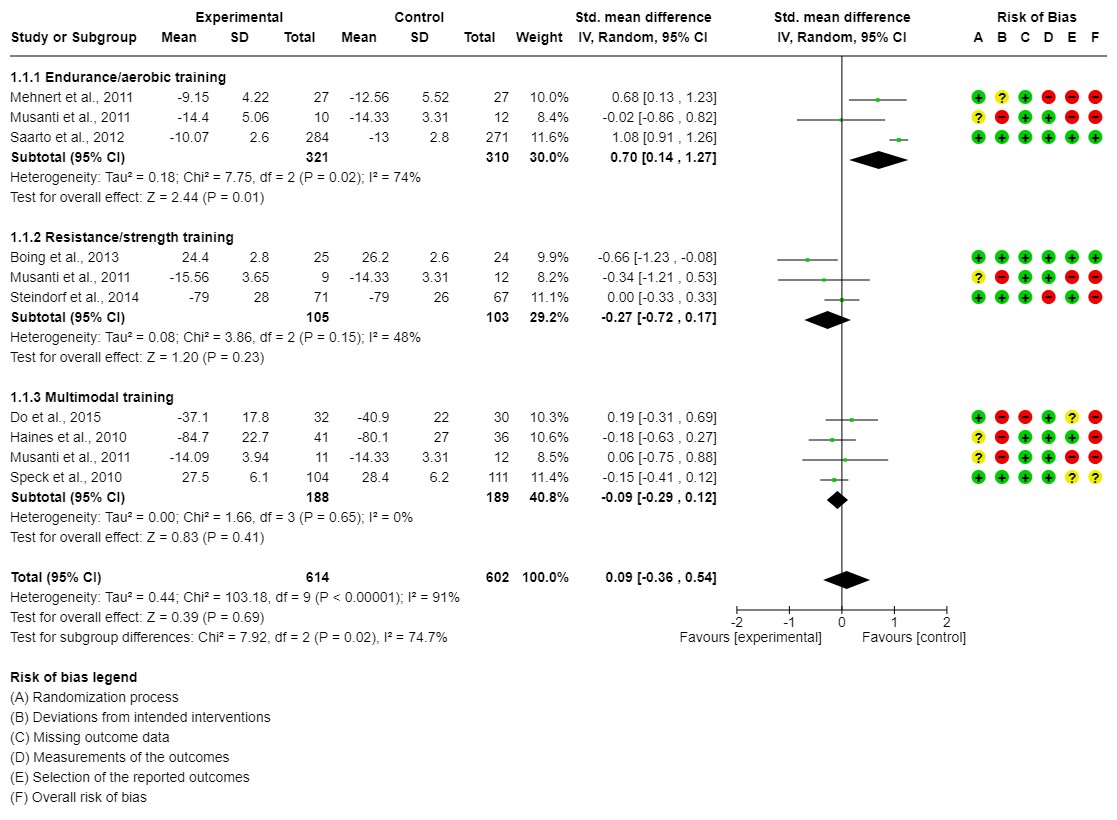


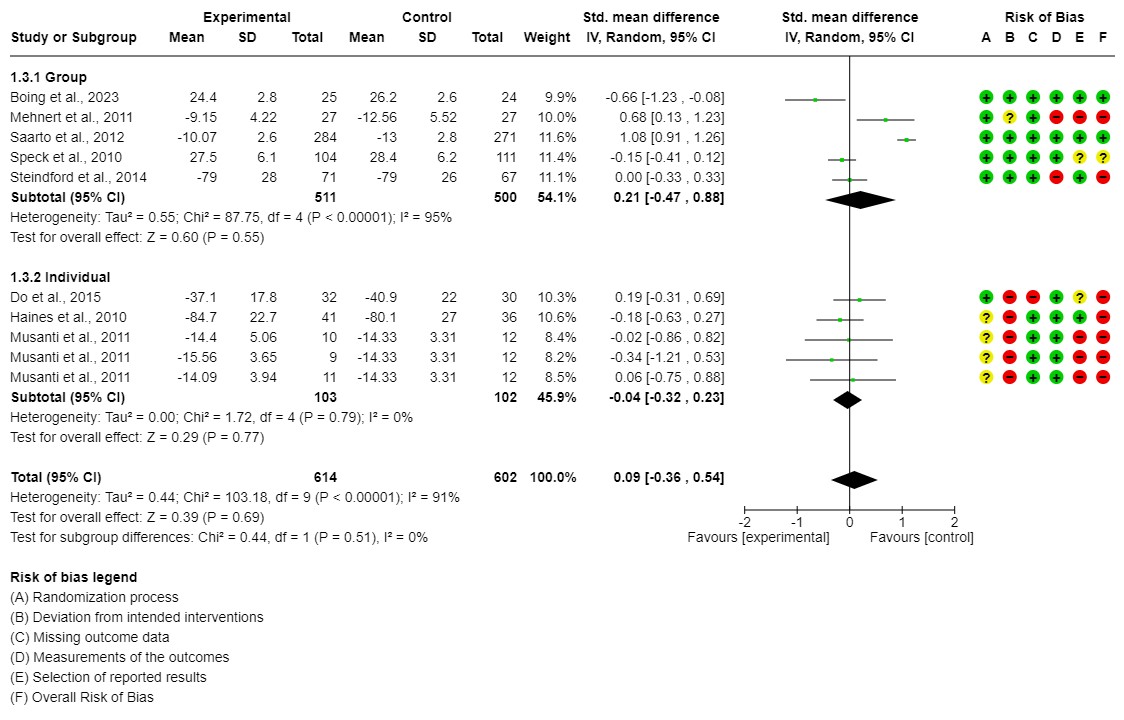
**Figure S9.** Subgroup analysis for self-perceived body image (group vs. individual).

**Figure S10.** Subgroup analysis for self-perceived body image (exercise supervision).


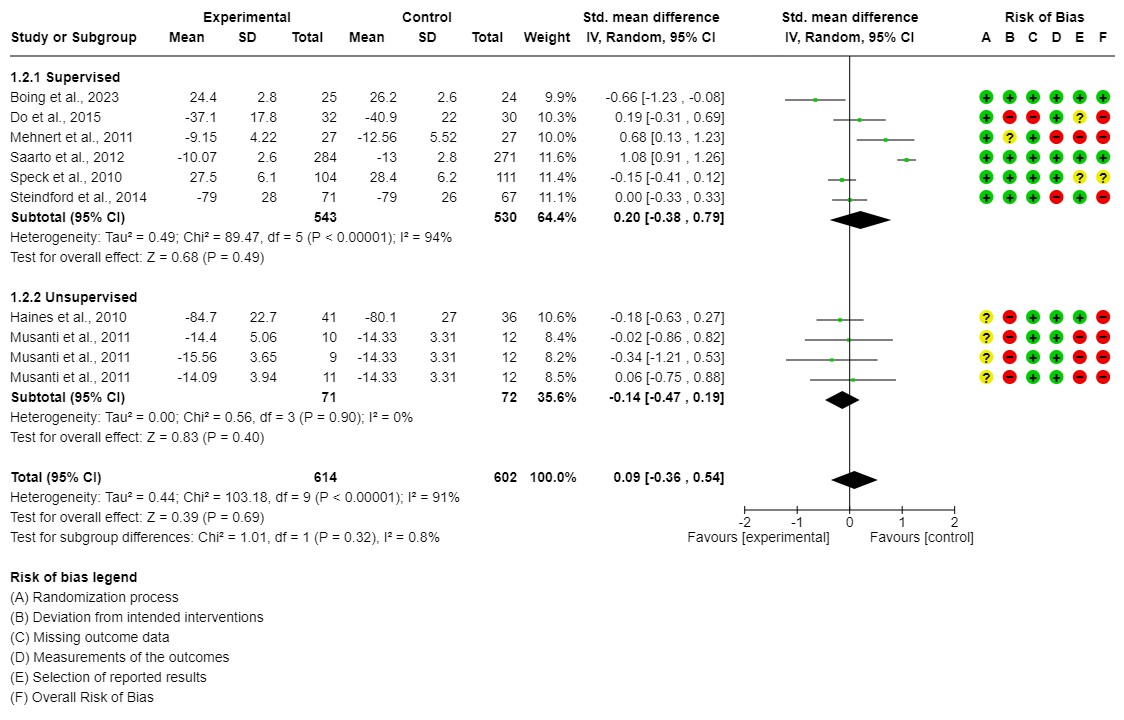


**Figure S11.** Subgroup analysis for self-perceived body image (risk of bias).


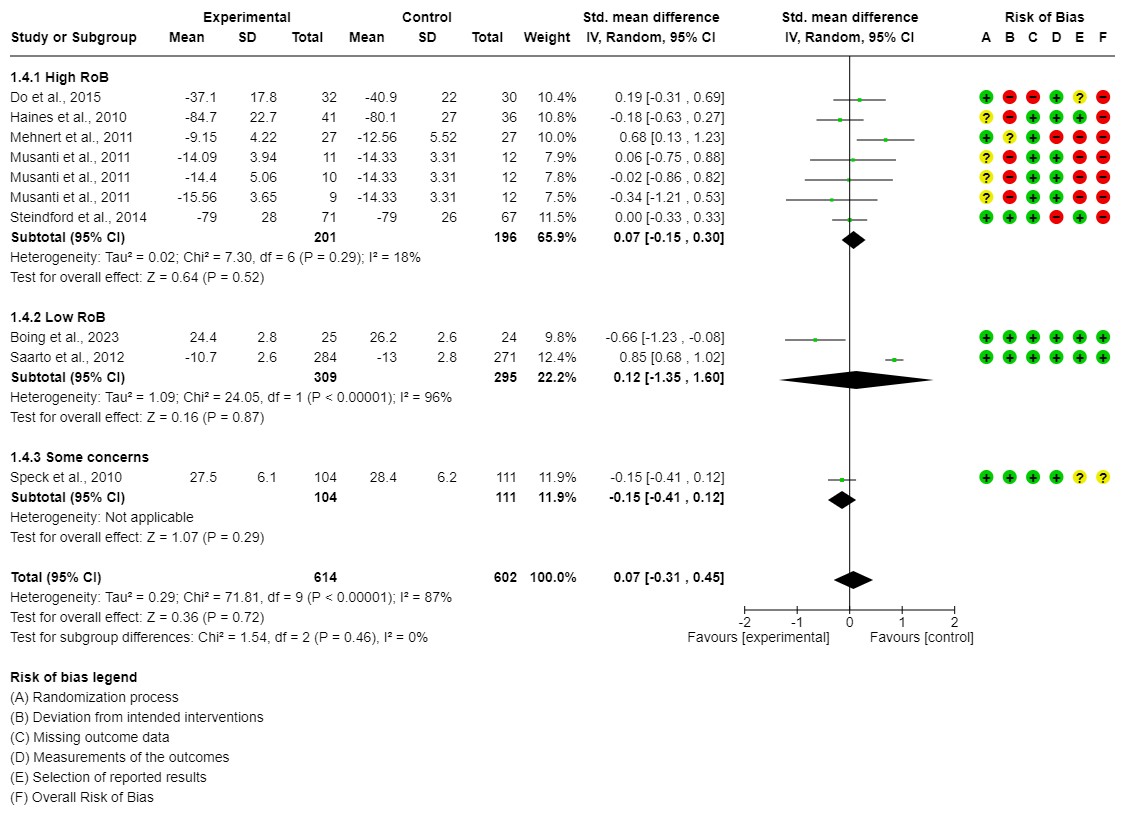


| **Body image** |  |  |
| --- | --- | --- |
| Covariate (*k*) | Coefficient β (95%CI) ^1^ | p-value |
| Age (10) | 0.01 (-0.33 to 0.34) | 0.95 |
| Sample size (10) | -0.01 (-0.01 to -0.004) | **<0.001*** |
| Minutes of experimental interventions (8) | 0.01 (-0.05 to 0.07) | 0.66 |
| Number of experimental sessions (8) | -0.02 (-0.03 to -0.004) | **0.01*** |
| Weeks of intervention (10) | -0.04 (-0.08 to -0.03) | **0.04*** |
| ^1^Mixed model effect-based meta-regression; Only moderators included in at least three studies were assessed; Redundant moderators were avoided; *k*: number of studies analyzed; * statistical significance (bold) p<0.05 | | |

**Table S12.** Univariate meta-regression analysis of covariates of the meta-analysis of body image score change.

**Figure S13.** Meta-regression plot for number of sessions and body image.


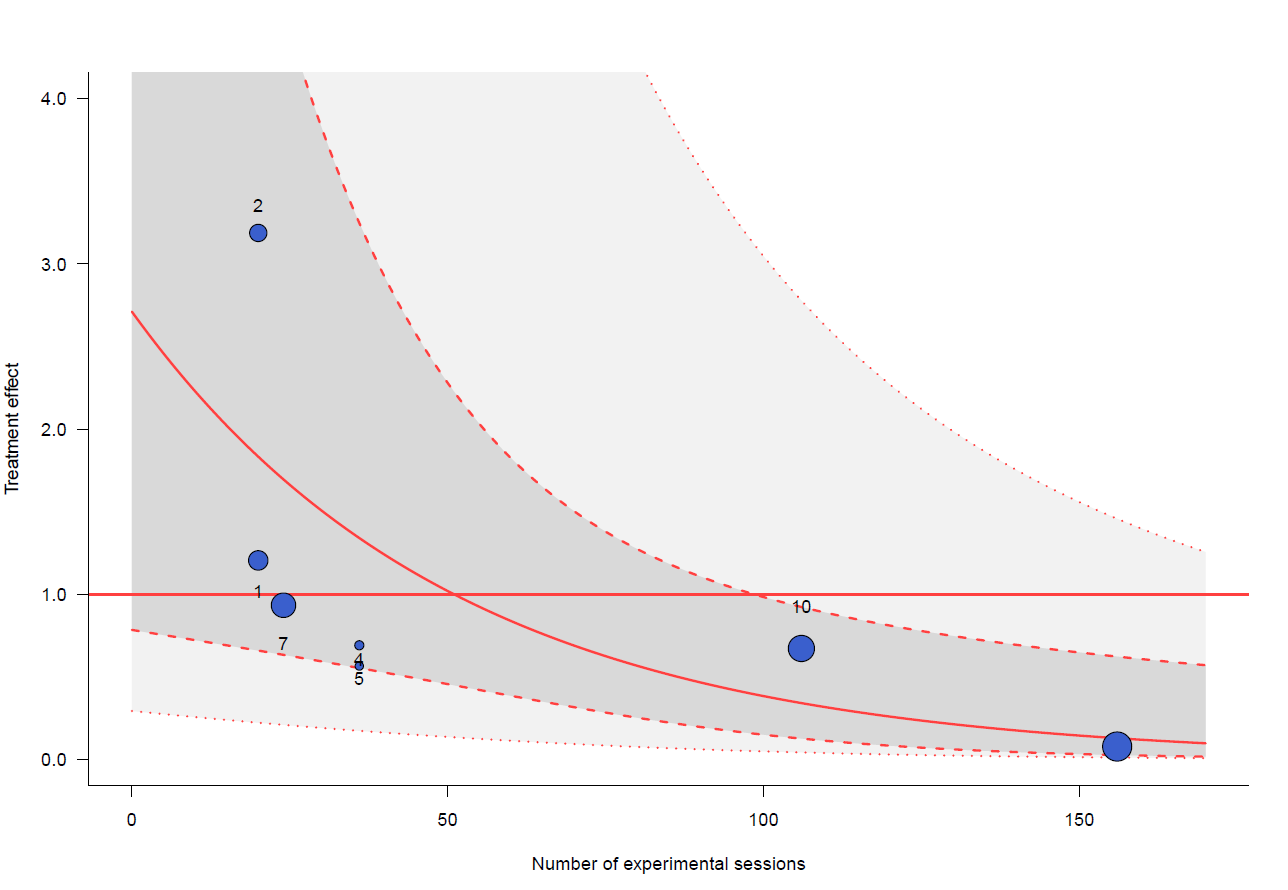


**Figure S14.** Meta-regression plot for sample size and body image.


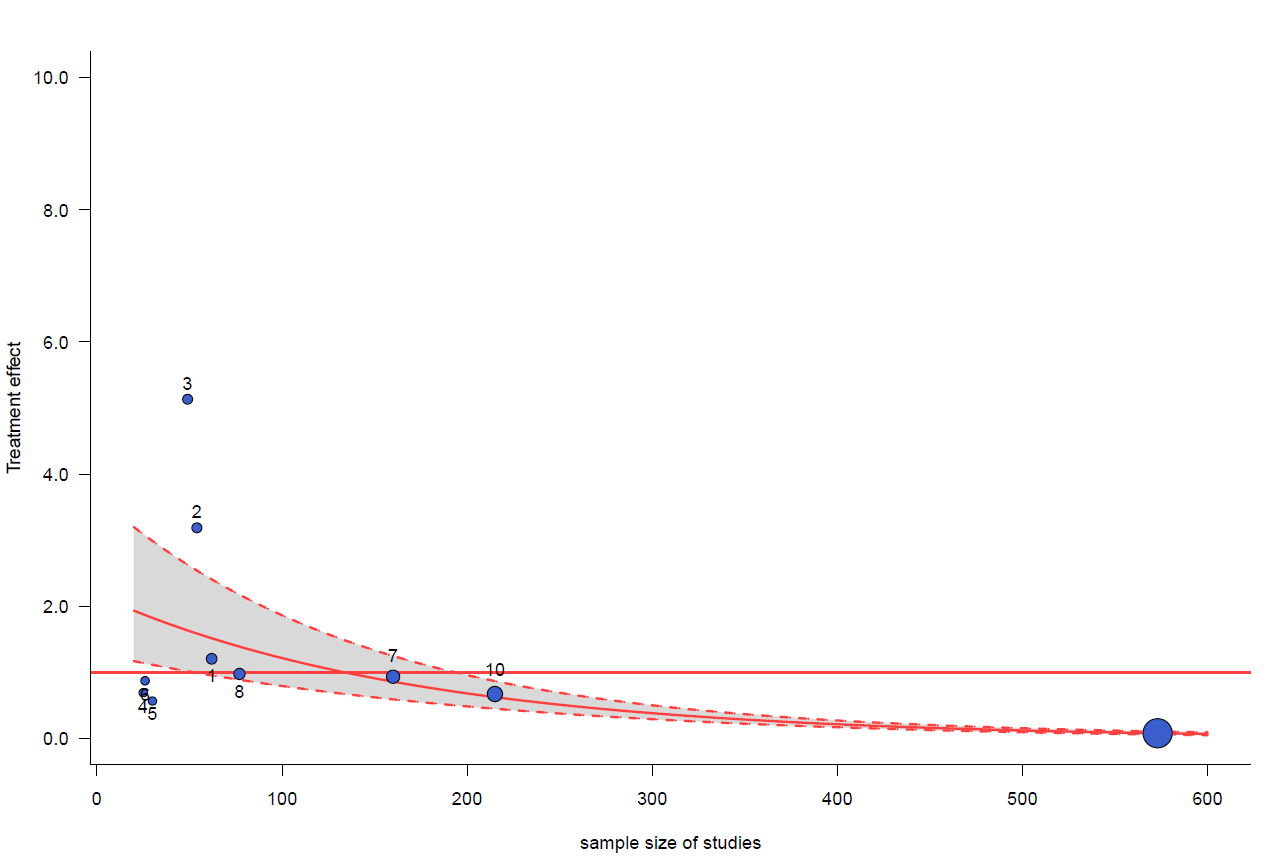


**Figure S15.** Meta-regression plot for weeks of intervention and body image.


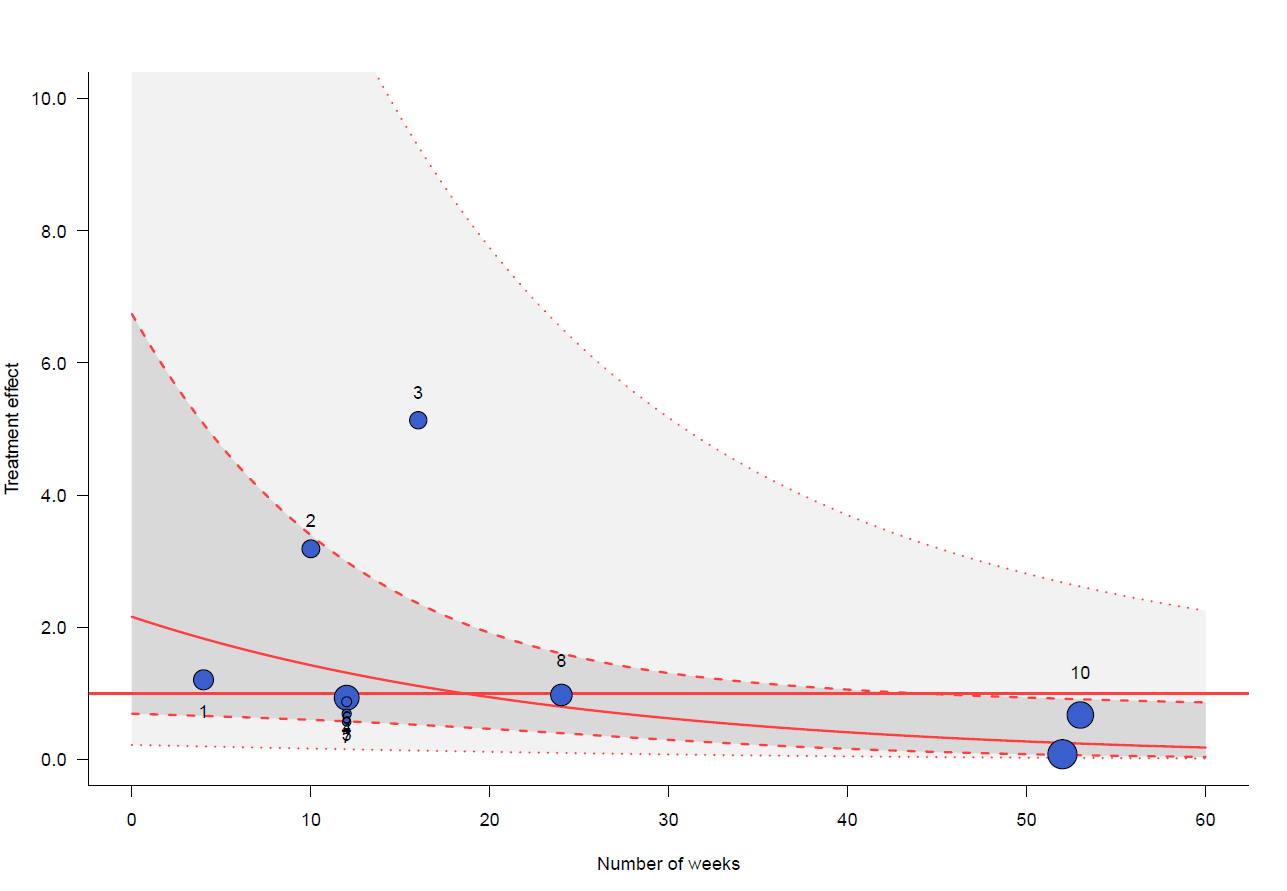


**Figure S16.** Sensitivity analysis for self-esteem outcome.


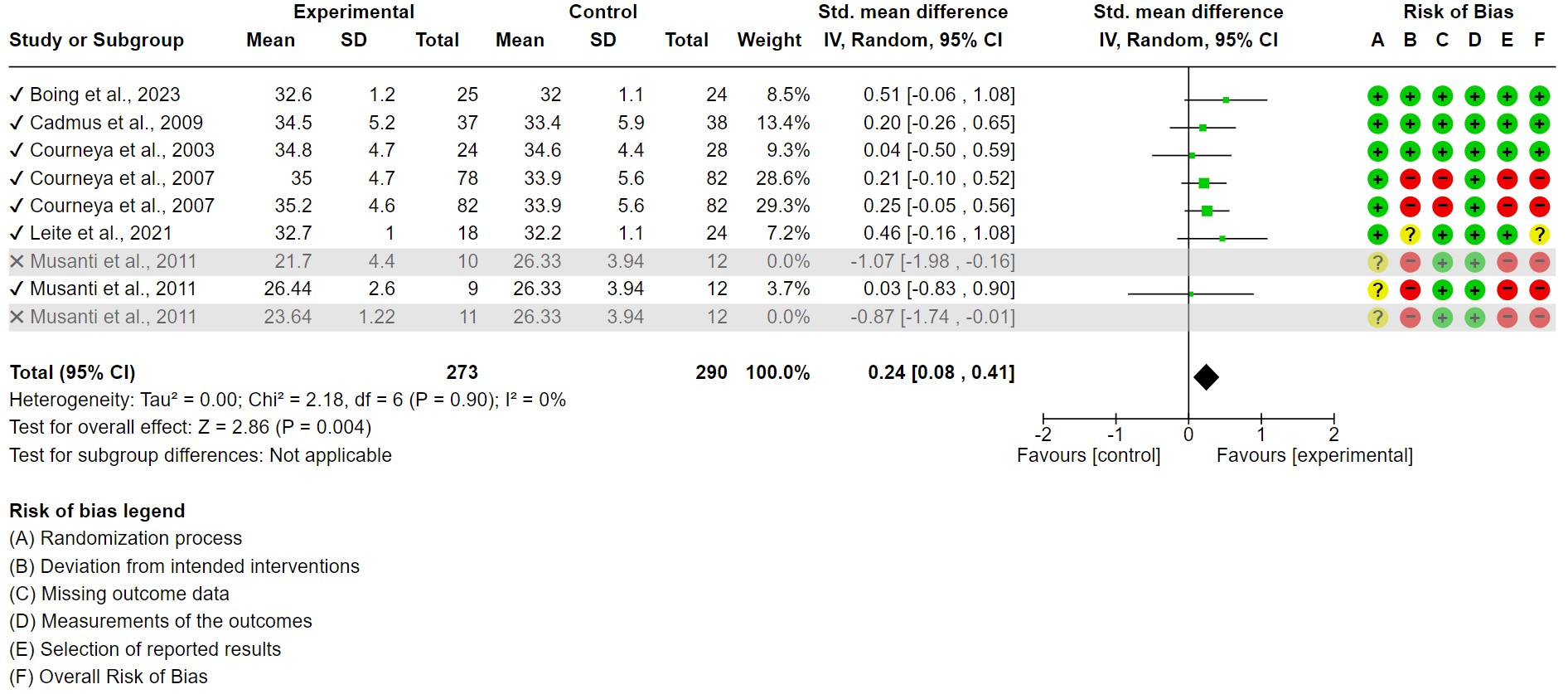


| Subgroup (number of arms of studies meta-analyzed) | SMD (95% CI) | I^2^ | Test for between subgroup differences  Chi^2^ (p-value)* |
| --- | --- | --- | --- |
| **Self steem (9)** |  |  |  |
| *Type of exercise*  Endurance/aerobic (4)  Resistance/strength (4)  Multimodal (1) | 0.00 (-0.39 to 0.39)  0.31 (0.07 to 0.55)  -0.87 (-1.74 to -0.01) | 58%  0%  NA | **7.70 (p=0.02)*** |
| *Exercise interventions in group*  Yes (2)  No (7) | 0.49 (0.07 to 0.91)  -0.01 (-0.29 to 0.28) | 0%  54% | 3.64 (p=0.06) |
| *Supervised sessions*  Yes (6)  No (3) | 0.25 (0.08 to 0.42)  -0.63 (-1.30 to 0.04) | 0%  42% | **6.29 (p=0.01)*** |
| *Risk of bias (RoB-2)*  High (5)  Low (3)  Some concerns (1) | -0.13 (0.56 to 0.30)  0.24 (-0.06 to 0.53)  0.46 (-0.16 to -1.08) | 69%  0%  NA | 2.93 (p=0.23) |
| *p-value <0.05 indicates between subgroups heterogeneity; NA: not applicable | | | |

**Table S17.** Subgroup meta-analyses for self-esteem.

**Figure S18**. Subgroup analysis for self-esteem (type of exercise).


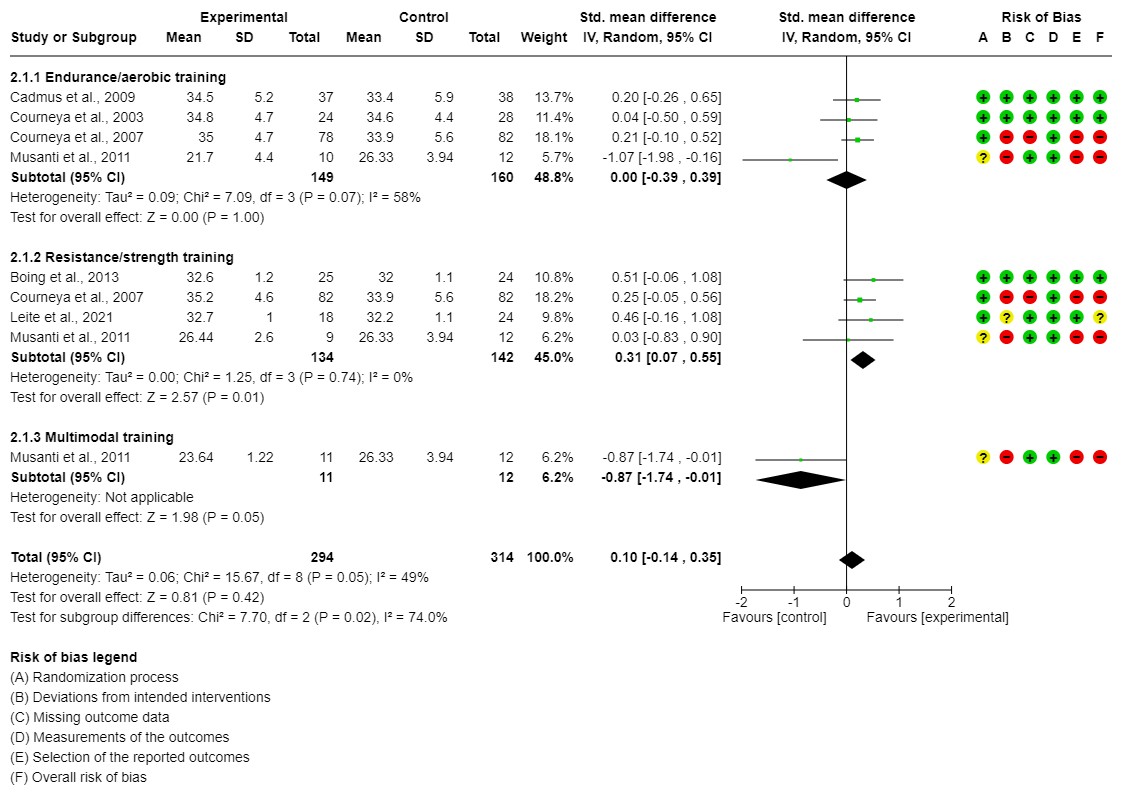


**Figure S19**. Subgroup analysis for self-esteem (group vs. individual).


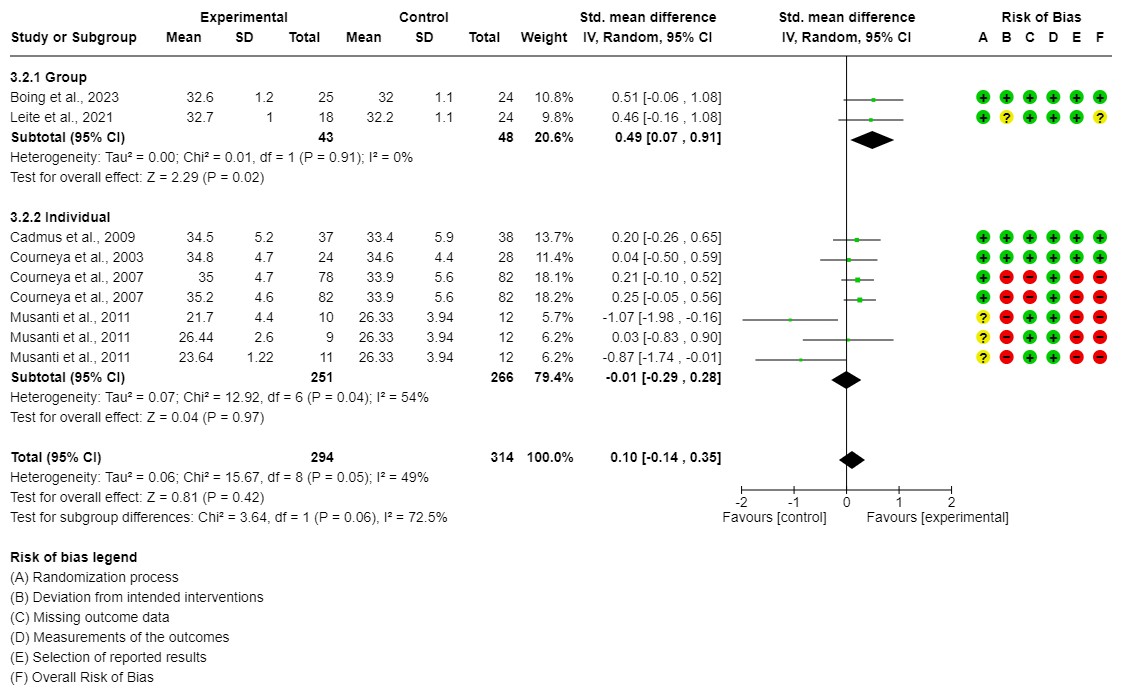


**Figure S20**. Subgroup analysis for self-esteem (exercise supervision).


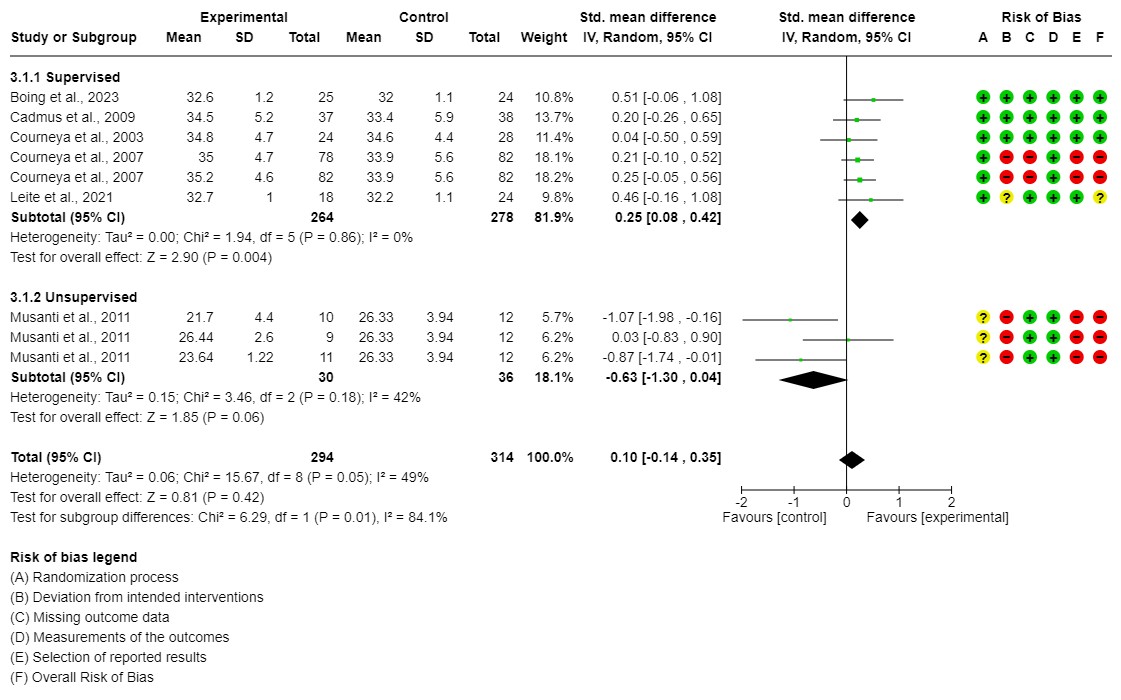


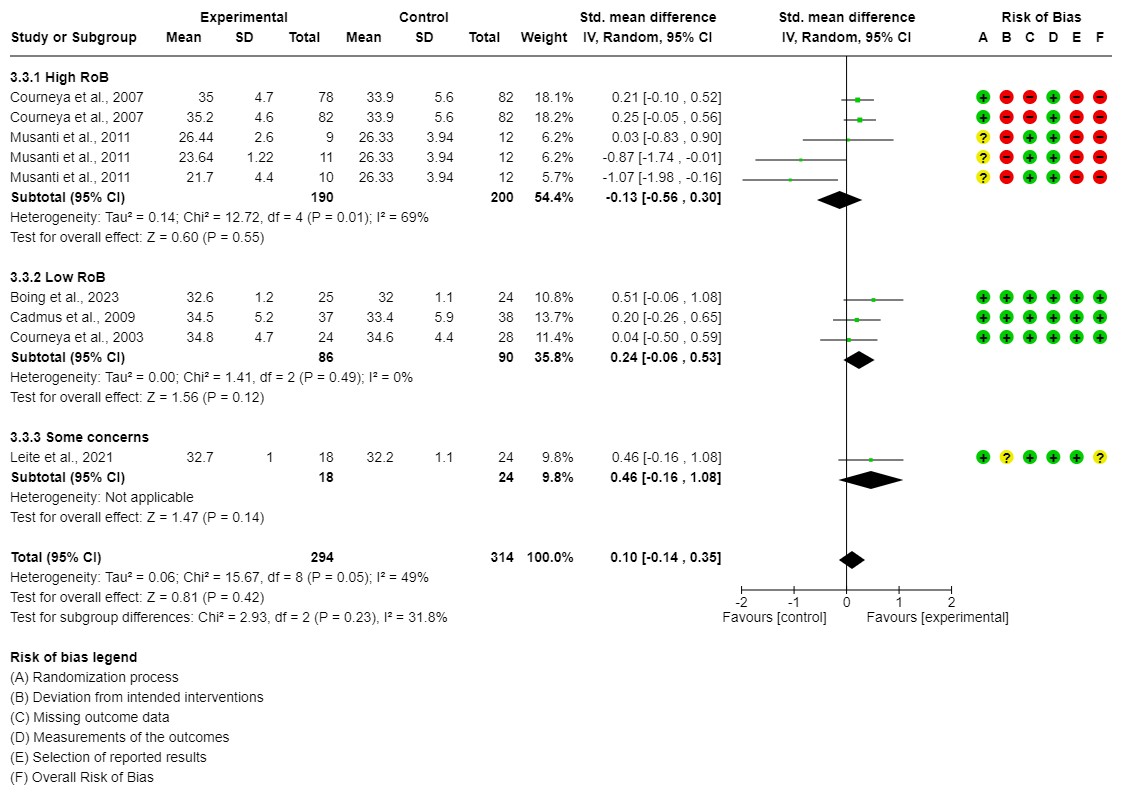
**Figure S21**. Subgroup analysis for self-esteem (risk of bias).

**Table S22.** Univariate meta-regression analysis of covariates of the meta-analysis of self-esteem score change.

| **Self-steem** |  |  |
| --- | --- | --- |
| Covariate (*k*) | Coefficient β (95%CI) ^1^ | p-value |
| Age (9) | -0.06 (-0.17 to 0.06) | 0.23 |
| Sample size (9) | 0.001 (-0.01 to 0.01) | 0.71 |
| Minutes of experimental interventions (6) | -0.03 (-0.08 to 0.02) | 0.3 |
| Number of experimental sessions (6) | 0.004 (-0.02 to 0.03) | 0.57 |
| Weeks of intervention (7) | -0.01 (-0.07 to 0.04) | 0.60 |
| ^1^Mixed model effect-based meta-regression; Only moderators included in at least three studies were assessed; Redundant moderators were avoided; *k*: number of studies analyzed; * statistical significance (bold) p<0.05; | | |
